# Why are critical event checklists not always used in the perioperative setting?: A retrospective survey

**DOI:** 10.1371/journal.pone.0314774

**Published:** 2025-02-28

**Authors:** Anna Clebone, P. Allan Klock Jr, Ellen Y. Choi, Avery Tung

**Affiliations:** Department of Anesthesia and Critical Care, University of Chicago, Chicago, Illinois, United States of America; Yale School of Medicine: Yale University School of Medicine, UNITED STATES OF AMERICA

## Abstract

**Introduction:**

During surgery and anesthesia, life-threatening critical events, including cardiac arrest, may occur. By facilitating recall of key management steps, suggesting diagnostic possibilities, and providing dose and drug information, cognitive aids may improve clinician performance during such events. In actual clinical practice, however, cognitive aids may be available but inconsistently used. One possibility explaining aid non-use during critical events is a lack of familiarity with how cognitive aids may be helpful. We hypothesized that introduction of critical event cognitive aids along with implementation of cognitive aid resources would change the quantitative incidence of cognitive aid use and qualitative reasons for aid non-use. We surveyed members of an academic anesthesia department before and after implementation of critical event cognitive aid resources.

**Methods:**

All anesthesia clinicians at a single academic medical center were surveyed.

Participants were surveyed both pre- and post-training with a focused program to introduce critical event cognitive aid resources. Incidences of and reasons for cognitive aid use and non-use were collected and analyzed. Survey responses were compared pre- and post-implementation.

**Results:**

The response rate was 64.5%. One-hundred eighty-five reasons for non-use were collected before the focused program and 149 after. Overall, 80% of clinicians had encountered at least one critical event during the study period and use of cognitive aids during all reported events was 7%. Six categories of reasons for non-use were identified: ‘Not Available’, ‘Not Needed’, ‘No Time’, ‘Another Person In Charge’, ‘Used In Another Way’, ‘No Reason Given’. After implementation, a decrease in the number of respondents who cited availability and who cited ‘another person running crisis,’ as reasons for non-use was observed (p < 0.001).

**Conclusions:**

Implementation of cognitive aids for critical events in an academic anesthesia environment improved the perception of cognitive aid availability and decreased the number of subjects who chose to not use the aid due to another person running the crisis response. Looking at the multiple reasons for cognitive aid non-use may guide implementation, training, and design.

## Introduction

Although anesthesia-related mortality is low, it is not zero.[1] Causes include rare but treatable critical events such as local anesthetic systemic toxicity and malignant hyperthermia.[1] A cognitive aid can assist with remembering critical, time-sensitive steps during rare events. Cognitive aids in the perioperative environment are often needed for specific reasons, such as to find a drug dose, consider a differential diagnosis, find more treatment ideas, or find specialized instructions such as defibrillation instructions.[2] This use is known as ‘sampling’ and is different than the use of cognitive aids in aviation, in which the items on a checklist are accessed in a sequential order.[2]

The reason for the different uses of checklists in medicine and aviation is due to the fundamental differences in these two environments. Aviation is a semi-closed environment, in which two pilots with the same training are working together to perform the same tasks. It is highly ordered and proceduralized, with a medium-to-high level of predictability, little variability across different aircraft of the same type leading to insignificant differences in equipment used daily.[2] The operating room is an open environment, with many people of different specialties accomplishing different tasks (surgery, anesthesia, nursing, technical), which is not proceduralized, often with a low level of predictability and differences in equipment from operating room to operating room.[2] Every patient and procedure is unique; every aircraft of a given make and model is similar.

For these reasons, we believe that checklist design, use, and implementation are more complex in medicine. During surgery and anesthesia, life-threatening critical events, including cardiac arrest, may occur.[3,4] By facilitating recall of key management steps, suggesting diagnostic possibilities, and providing dose and drug information, cognitive aids may improve clinician performance during such events.[5,6] In actual clinical practice, however, cognitive aids are inconsistently used. Barriers to checklist use include poor design (confusing wording, irrelevance, or redundancy), and a perception that the checklist is a waste of time.[7] Another possibility is a lack of familiarity with cognitive aid use and how such tools may be helpful. Among operating room technologists and nurses, a comprehensive implementation program effectively increased awareness of and familiarity with cognitive aids.[8] We hypothesized that the introduction of cognitive aids into a clinical anesthesia environment would change the incidence of cognitive aid use and reasons for aid use and non-use among anesthesia providers. To test our hypothesis, we surveyed members of an academic anesthesia department before and after cognitive aid implementation.

## Methods

The research components of this study were granted exempt status with approval for verbal consent by the University of Chicago Institutional Review Board, IRB 16-0718. In 2016 the hospital at which this research occurred had 805 licensed beds. In all specialties, it had 40 institutes and centers, approximately 850 attending physicians, approximately 2,500 nurses, and about 1,100 residents and fellows. The number of surgical cases and procedures, by American Society of Anesthesiologists (ASA) status is listed in Table 1. All members of the anesthesia department at this single large academic medical center were asked to complete a survey before and after implementation of a set of cognitive aids targeted at intraoperative critical events. In this survey, subjects were asked to recall events they had experienced during the past 3-6 months. Cognitive aid content was developed via an eight-member departmental task force and intended for use by all clinical anesthesia providers. The survey, administered in either paper or electronic form, (S1) included participant years of experience and current role and asked about recent experience with critical events, whether already existing cognitive aids were used, and reasons for use and non-use. Participants were given a taxonomy of critical events to choose from (Table 2). Already existing cognitive aids included a departmental aid for obstetric hemorrhage as well as aids with prior national implementation (e.g., malignant hyperthermia and advanced cardiovascular life support). The pre-implementation survey was administered from June 9 to July 30 2016, and the post-implementation survey was administered from March 9 2017 to October 10 2017. Verbal consent for these surveys was obtained and documented for each participant by the researcher performing data collection.

**Table 1:** Case types by American Society of Anesthesiologists (ASA) Classification During Study Years at Study Hospital.

Variable	Year 2016	Year 2017
ASA^a^	Number of Cases	Number of Cases
1	3,242	3,359
2	11,008	11,007
3	12,723	14,715
4	2,173	2,633
5	88	87
6	16	17
Total	29,250	31,818

^a^ASA Classification =  American Society of Anesthesiologists Classification [9].1. “Healthy person.2. Mild systemic disease.3. Severe systemic disease.4. Severe systemic disease that is a constant threat to life.5. A moribund person who is not expected to survive without the operation.6. A declared brain-dead person whose organs are being removed for donor purposes.”

**Table 2:** Taxonomy of Critical Events.

Events
Anaphylaxis^a^
Air Embolism^a^
Bradycardia^a^
Cardiac Arrest^a^
Unexpected Difficult Airway
Massive Hemorrhage^a^
Fire
Hyperkalemia^a^
Hypertension
Hypotension
Hypoxia
Increased Intracranial Pressure^a^
LAST – Local Anesthetic Systemic Toxicity^a^
MH – Malignant Hyperthermia^a^
Myocardial Ischemia
Tension Pneumothorax^a^
Transfusion Reaction^a^
Other

^a^Event Part of Set Cognitive Aids Implemented for this Research (S2).

### Implementation

After cognitive aids were developed, an implementation plan was initiated. Cognitive aids were available as laminated 8.5 X 11-inch cards in the top drawer of each anesthesia machine, attached by a metal O-ring in the upper left-hand corner so they could be easily flipped from page to page and lie flat when flipped. A sticker with a red border was placed on each drawer that said ‘ADULT Crisis Quick Reference Guide and Cognitive Aid.’ These aids were intended for primary use by the anesthesiology team, however, it was expected that the anesthesiology team leader would direct others in the room to take actions based on the aids as needed.

Implementation occurred from August 2016 to February 2017 and consisted of a lecture based on existing training protocols given to the entire department (S3),[10] and monthly resident lectures that were open to the entire department[11] as well as posting of the cognitive aids in large format in a common area. Specific points of emphasis included potential reasons for cognitive aid non-use, how cognitive processing abilities decrease in emergent, time-pressured environments, and specific examples of cases demonstrating the utility of critical event cognitive aids.

### Response assessment

The critical event type was indicated in the survey responses and compared between pre- and post-cognitive aid implementation.

Narrative responses from both pre- and post-implementation surveys were analyzed qualitatively by a study author (AC) and researcher (KJR) as follows: First, mutually exclusive categories of modifiable and non-modifiable reasons for cognitive aid non-use were developed from survey data by author AC (Table 3). Reasons for cognitive aid non-use were then independently classified into those categories. Discrepancies in classification occurred for 39 out of 334 items (12%). All 39 discrepancies were eventually resolved through discussion between AC and KJR.

**Table 3:** Reasons for Cognitive Aid Non-Use.

Reason	Initial (Before Implementation)*	Follow-Up (After Implementation)*	Z	p
A = ‘not available’ ^a^	50	17	5.74	0.001
N = ‘not needed’ ^b^	60	57	1.73	0.083
T = ‘no time’ ^c^	14	19	2.24	0.025
P = ‘another person running crisis’ ^d^	25	13	3.46	0.001
H = ‘used in another way’ ^e^	15	16	1	0.317
O = no reason given	21	27	2.45	0.014
Total	185	149	334*	

*For some participants >  1 reason stated for cognitive aid Non-use; for some participants no reason was stated.

+ Sign Test. P-value (two-tailed) <  0.008 considered as significant with Bonferroni correction for multiple outcomes.

^a^A =  ‘not available:’ Did not have cognitive aid/forgot about cognitive aid.

^b^N =  ‘not needed:’ Knew what to do already/cognitive aid would not help.

^c^T = ‘no time:’ Too busy/completing other tasks.

^d^P =  ‘another person running crisis:’ Another person knew what to do.

^e^H =  ‘used in another way:’ Did not pull out cognitive aid but did use cognitive aid that was previously memorized or known/someone else using a cognitive aid/used a cognitive aid later on.

### Statistics

Descriptive statistics were used for the overall number of participants encountering at least one critical event and the number of participants reporting aid non-use. Pre- and post-implementation groups were quantitatively compared for each category of reason for cognitive aid non-use. The overall number of times that a specific category of reason was used was also compared between types of reported events. The Social Sciences Calculator and Stats Blue were used for statistical analysis. A Shapiro-Wilk test was used to determine if the data existed in a normal distribution, although a non-normal distribution was expected for this categorical data. A Sign test was used for comparison of categorical values. The number of reasons given for cognitive aid non-use pre- and post-implementation was similar, although not identical, therefore, this number was averaged to use a statistical test that required paired values.

We performed several logistic regression analyses: 1) To examine the effect of the level of training on cognitive aid use and 2) To examine the effect of type of critical event (e.g., anaphylaxis, air embolus, etc., listed in Table 2) and reason for aid use on pre- or post-implementation status.

## Results

Rates of response and critical event encounters are reported in Table 4. Participants reported cognitive aid non-use in 94% of events pre-implementation and 91% of events post-implementation. Narrative reasons for cognitive aid non-use were divided into six categories: A = ’not available,’ N =  ‘not needed,’ T =  ‘no time,’ P =  ‘another person running crisis,’ H = ‘used in another way,’ O =  no reason given. (Table 3) This data was not normally distributed. The number of narrative statements corresponding to each category did not differ between pre- and post-aid implementation for all categories except for reason A =  ‘not available,’ p < 0.001 and reason P =  ‘another person running crisis,’ p < 0.001. See ‘S4’ for all narrative reasons for non-use. A Bonferroni correction was used to account for multiple outcomes, and p < 0.008 was considered as significant for each of multiple primary hypotheses. (Table 3)

**Table 4:** Retrospective Survey of Entire University of Chicago Department of Anesthesia and Critical Care (attending physicians, residents, fellows, nurse anesthetists).

Variable	Initial	Follow-Up (After Cognitive Aid Implementation Training)	Overall
Number of Subjects	93	90	
Total Number of Clinicians in the Department of Anesthesia ^a^	158	129	
Response Rate %^b^	59	70	64.5
Intern ^c^	2	0	2
Clinical Anesthesia Resident Year 1^c^	22	14	36
Clinical Anesthesia Resident Year 2^c^	6	10	16
Clinical Anesthesia Resident Year 3^c^	9	8	17
Fellow ^c^^,^^d^	4	1	5
Nurse Anesthetist ^c^	16	19	35
Attending Physician ^c^^, e^	31	38	69
Did Not Specify	2	0	2
Any Critical Event Encounter over past 3-6 months %	80	79	79.5
Number of critical events encountered over past 3-6 months	180	155	335
Number of critical events for which ^a^ cognitive aid was used	12	15	26
Events for which ^a^ critical event cognitive aid was used %	6	9	7.5

^a^The department size fluctuated in-between surveys, largely because incoming and outgoing residents overlapped for the initial survey.

^b^Percentage of entire department that responded.

^c^An independent means t-test showed no difference in training or professional level between the initial and follow-up groups, t =  -1.30623; p =  0.193.

^d^A fellow has completed their primary anesthesiology residency and is completing an additional year of subspecialty training.

^e^An attending physician has fully completed their training in anesthesiology and is qualified to perform anesthesiology independently, in a supervisory role, and as a perioperative consultant physician.

Overall, cognitive aids were used in only 7% of described critical events. (Table 4) and in 30% of events for which the cognitive aid was accessed, the clinician explicitly described this access as occurring after initial critical steps were performed. (S5) There was no relationship between the level of training and cognitive aid use, OR 0.86; 95% CI 0.71,1.04; p = 0.119.

Overall, implementation status did not influence the types of critical events reported, OR 1; 95% CI 0.96,1.04; p = 1.00 however, implementation status did correlate with reasons for cognitive aid non-use, OR 1.17; 95% CI 1.03,1.34; p = 0.02. (Table 5)

**Table 5:** Reasons for Cognitive Aid Non-use by Event.

Event	A = not available			N = did not need/knew what to do			T = no time			P = another person running			H = used a cognitive aid in another way			O = no reason given		
	Pre	Post	Total	Pre	Post	Total	Pre	Post	Total	Pre	Post	Total	Pre	Post	Total	Pre	Post	Total
Air Embolus	1		1					1	1									
Anaphylaxis	5	2	7	2	2	4					1	1		1	1	1		1
Bradycardia	5		5	4	9	13	1		1				1	1	2		2	2
Cardiac Arrest	6	2	8	4	4	8		3	3	7	3	10	3		3	1		1
Hyperkalemia	1		1	5	2	7					1	1		1	1			
Hypertension		1	1							1		1						
Hypotension	5	3	8	5	11	16	1	3	4	1		1		1	1		1	1
Hypoxia	6	1	7	10	9	19	4	3	7	4		4	4	1	5	2		2
Increased Intercranial Pressure		1	1		1	1				1		1						
Malignant Hyperthermia	1		1	1		1				1		1						
Massive Hemorrhage	6	1	7	12	7	19		3	3	6	3	9		1	1	1	4	5
Myocardial Ischemia	1	1	2	1	1	2					3	3	1		1			
Pulmonary Embolus	1		1	1		1	1		1	1		1	1		1	1		1
Pulmonary Hypertension							1		1	1		1						
Tachycardia				1		1												
Tension Pneumothorax				1		1												
Unexpected Difficult Airway	8	3	11	7	9	16	3	6	9	1	1	2	5	10	15	4		4
Other	4	2	6	6	2	8	3		3	1	1	2				11	20	31
TOTALS	50	17	67	60	57	117	14	19	33	25	13	38	15	16	31	21	27	48

## Discussion

We administered a pre- and post-implementation retrospective survey of reasons for cognitive aid use and non-use in an academic anesthesia department. We found that implementation of critical event cognitive aids along with a focused program to introduce clinicians to cognitive aid resources decreased the perception that cognitive aids were unavailable and decreased the number of incidences of aid non-use because of a perception that another person was running the crisis. 80% of respondents had encountered a critical event during the study period but cognitive aids were only used in 7% of events. Cognitive aids were not used more frequently after implementation.

Our results are consistent with existing literature finding inconsistent use of cognitive aids. In a 2019 simulation study, fewer than half of participants chose to use an emergency manual despite formal practice-wide critical event cognitive aid implementation.[12] Our study found an even higher rate of non-use, and, in addition, identified perceived reasons for aid non-use. In addition, we observed that even after introducing cognitive aids and deploying a program to familiarize clinicians with their use and rationale, only two reasons for aid non-use changed.

The mechanisms underlying cognitive aid non-use are incompletely understood. In our survey, the most common reason was “not needed/know what to do”, followed by “no time.” One possibility is that most cognitive aids are not designed to be user-friendly. Analyzing how tools are used and adapting the tool to the user rather than making the user adapt to the tool may increase success. For example, anesthesia clinicians may “sample” a cognitive aid for specific information rather than using the aid as a step-by-step “linear” instruction set.[10] Aids designed for such a linear approach may not be as popular as aids designed for a “sampling” type of access. We have previously observed that cognitive aid design may affect the perceived “usability”[13] and speed of access[14] of cognitive aids. Another reason for non-use may be that clinicians are already familiar with the appropriate response to critical events and do not feel they need a cognitive aid to effectively manage a critical event. This may explain why different reasons for non-use were found for different event aids (e.g., anaphylaxis versus hyperkalemia).

Our study has implications for the use of cognitive aids in anesthesia practice. We observed that even an organized rollout of cognitive aids and a focused program detailing their potential utility did not meaningfully increase their use among anesthesia clinicians. Our work suggests that introducing cognitive aids to a clinical practice may not be straightforward and that a prolonged, focused rollout may be needed. Taken together with our previous work, our findings also raise the possibility that optimizing the perceived utility of cognitive aids through improved design may facilitate aid use during critical events in anesthesia.

Our data may also guide future modifications of our program for familiarizing clinicians with cognitive aids. Cognitive aid implementation during this study was brief, and primarily targeted aid availability. Future implementation, training, and design efforts may be improved by addressing the reasons for cognitive aid non-use described by survey respondents. In 35% of surveys, participants noted that the aid was not needed, for example because they ‘knew what to do already’ or that they thought the ‘cognitive aid would not help.’ Formatting cognitive aid content to more closely align with clinician use improves usability[13] and speed of access,[13] and may improve aid use. In surveys, participants reported that they did not need a cognitive aid because ‘another person was running the crisis”, and 9% of survey participants noted that they did not have enough time to access the cognitive aid. These challenges may both be addressable via technological strategies to allow all participants in critical event management to view the cognitive aid, for example, on a large screen.

Our study has limitations. Because we surveyed participants about critical events that occurred in the past, the nature of the critical event and reasons for aid non-use may have been distorted by recall bias. In addition, our study was performed at a single academic center without a history of extensive cognitive aid use. Reasons for non-use may differ in another perioperative environment. Our choice of cognitive aids was also limited to specific critical events relevant to local practice. However, many participants reported critical events such as hypotension and hypoxia for which a cognitive aid had not been created. Better matching of cognitive aids to real world critical event occurrences may also have changed our study results. There was no relationship between the level of training and cognitive aid use. This is likely because of the high level of variability of critical events encountered in the operating room setting and the limited time period studied. A larger number of participants studied, or, conversely, a more limited set of critical events, may have shown a relationship between level of training and cognitive aid use.

This data was collected in 2016 and 2017, and fast-paced advancements in medical technology and practices have occurred since that time. However, major changes have not been made in the discrete steps needed for clinical management of critical events in anesthesiology, which is the subject of the research presented in this manuscript.

To present a comprehensive picture of critical event cognitive aid use and non-use, all members of the department were studied, which included members of the task force that developed the cognitive aids. It is possible that members of the task force answered differently due to this additional exposure to cognitive aids. Nevertheless, the small number of department members on the task force (8 individuals) makes the impact of this exposure on the results likely to be minimal. Furthermore, other cognitive aids, such as that for malignant hyperthermia, were in longstanding use in the department already, which further dilutes the effect of this additional cognitive aid exposure from assisting with aid development.

Additionally, the number of department members varied between the two surveys, as outgoing third-year residents and incoming first-year residents overlap in their training and other staffing fluctuations occur.

To achieve more honest responses and in accordance with the approval from our institutional review board, subjects were permitted to be anonymous. Therefore, although many of the same individuals were questioned before and after implementation, it is unknown how much overlap occurred.

Another limitation was the period for recall of events studied, 3-6 months. It is possible that different results would have been found if the recall period was shorter or longer.

In conclusion, we found that a formal, planned introduction of critical event cognitive aids to an academic anesthesia department did not increase cognitive aid use or change the reasons for cognitive aid non-use. Our findings suggest that implementing a cognitive aid program into anesthesia practice is a complex undertaking and that immediate acceptance is unlikely. Two common reasons in our study: “already know what to do” and “someone else is running the critical event response” raise the possibility that designing cognitive aids to be more aligned with clinician needs may improve aid use, as may strategies to make cognitive aids available to more members of the care team. More work is needed to better understand reasons for cognitive aid non-use and identify strategies for greater acceptance and usage of cognitive aids in anesthesia care.

## Supporting information

S1 File Survey. (DOCX)

S2 File Cognitive Aids 2016. Email corresponding author for a copy of updated aids.
(PDF)

S3 File Grand Rounds Lecture. (PDF)

S4 File Narrative Reasons for Aid Non-Use. (DOCX)

S5 File Narrative Reasons for Aid Use. (DOCX)
