# Supplementary material for: Why are critical event checklists not always used in the perioperative setting?: A retrospective survey
Source: PLoS One. 2025 Feb 28;20(2):e0314774. doi: 10.1371/journal.pone.0314774 (PMC11870359; doi:10.1371/journal.pone.0314774)
Supplement: S1 File — (DOCX) [file pone.0314774.s001.docx]

**Survey Questions**

Current Position

1. Please think about the critical events you have seen lately. How many critical events have you seen in the operating room, post-operative care unit, or immediately postoperatively in the ICU, that you remember well, in the past 6 months??
2. Please indicate the critical event cognitive aids or guides you are familiar with:

Malignant Hyperthermia

Local Anesthetic Systemic Toxicity

Ariadne Labs Crisis Checklist

Society for Pediatric Anesthesia Crisis Checklist

Stanford Emergency Manual

Other

None

1. Which other critical events cognitive aids or guides are you familiar with?

**Critical event questions (repeated for each event)**

1. 1st Critical Event: 1) Check the box(es) corresponding to the relevant critical event (selection provided)

🞏 Anaphylaxis 🞏 Fire 🞏 LAST

🞏 Air Embolus 🞏 Hyperkalemia 🞏 MH

🞏 Pulmonary Embolus 🞏 Hypertension 🞏 MI

🞏 Bradycardia 🞏 Hypotension 🞏 Massive Hemorrhage

🞏 Cardiac Arrest 🞏 Hypoxia 🞏 Tension PTX

🞏 Unexpected difficult Airway 🞏 Increased ICP 🞏 Transfusion Rxn

🞏 Other (write in) __________________________

1. What type of critical event occurred (1st critical event):
2. What was your role in the case? (check all that are applicable) (1st critical event)
3. We are trying to identify situations in which a cognitive aid might be useful. For this event, did you use a critical events cognitive aid DURING the event? (1st critical event)
4. If you did not use a cognitive aid: Why not? (1st critical event)
5. If you did use a cognitive aid - which ONE(s)? (1st critical event)
6. What prompted you to pick up the cognitive aid? (1st critical event)
7. At what point in the event did you pick up the cognitive aid? (1st critical event)
8. What was useful about the cognitive aid? (1st critical event)
9. What was NOT useful about the cognitive aid? (1st critical event)
10. What was NOT useful about the cognitive aid? (1st critical event)
11. Is there any other information you would like to share with us about this event or cognitive aid use?

(slight changes in format for second survey)
