# Supplementary material for: Why are critical event checklists not always used in the perioperative setting?: A retrospective survey
Source: PLoS One. 2025 Feb 28;20(2):e0314774. doi: 10.1371/journal.pone.0314774 (PMC11870359; doi:10.1371/journal.pone.0314774)
Supplement: S2 File — Email corresponding author for a copy of updated aids. (PDF) [file pone.0314774.s002.pdf]

THE UNIVERSITY OF CHICAGO

Department of Anesthesia and Critical Care

ADULT Crisis Quick Reference Guide and Cognitive Aid

1	Air Embolism
2	Anaphylaxis
3	Bradycardia/Pacing
4	Cardiac Arrest
5	Hyperkalemia
6	Increased Intracranial Pressure
7	Local Anesthetic Systemic Toxicity
8	Malignant Hyperthermia
9	Massive Hemorrhage/Transfusion
10	Tension pneumothorax
11	Transfusion Reactions
12-15	MAPS of DACC Locations

Call attending

Call for help!

Anest. Coordinator

dial - 64470, page 4470

Notify surgeon

Revision July 19, 2016

This reference is meant to serve as a memory aid and is not intended to proscribe care. It does ***not*** replace your clinical judgment.

Adapted for local use from resources authored by the Society for Pediatric Anesthesia, Ariadne Labs, ASRA, MHAUS, and NASA

1 Air Embolus ADULT

VERIFY DX – STABILIZE PATIENT

- Verify Air Embolus: ↓ $EtCO_2$ ↓ SaO_2 ↓ BP , clinical diagnosis
  - More likely if surgical site above heart or air in IV line
- Notify surgeon
- Give 100% O_2, evaluate ventilation
- Stop nitrous oxide, if hypotensive stop anesthetic agents

TREATMENT

Cause	Action
Air entrainment from surgical site	▪ Tell surgeon to flood wound with irrigation *and* turn off all pressurized gas sources, e.g. laparoscope, endoscope<br>▪ Lower surgical site below level of heart (if possible)<br>▪ If air entrainment from head or neck, compress jugular veins intermittently<br>▪ Perform valsalva using hand ventilation
Air entrainment from IV lines	▪ Check for open venous lines or air in IV tubing
Hypotension (can be from air in heart)	▪ Epinephrine 50 mcg-1 mg IV bolus or 0.02-1 MICROgrams/kg/min IV<br>▪ Norepinephrine 0.05-2 MICROgrams/kg/min IV<br>▪ Chest compressions 100/min to force air through lock, even if not in cardiac arrest
**Partial Differential**	
Embolus: Fat, thrombotic, cement, amniotic fluid	▪ If laparoscopic, de-sufflate<br>▪ If not laparoscopic, stop surgery
Anaphylaxis	▪ See CHKLST 2 (Anaphylaxis)
Local Anesthesia Systemic Toxicity	▪ See CHKLST 7 (LAST)
Cardiac Ischemia	▪ Morphine, oxygen, aspirin, NTG, beta-blockers, heparin

DRUG / DOSAGE SUMMARY

- **Epinephrine** 50 mcg-1 mg IV bolus or 0.02-1 MICROgrams/kg/min IV
- **Norepinephrine** 0.05-2 MICROgrams/kg/min IV

CRISIS MANAGEMENT (If Severe)

- Notify surgeon, call for help and code cart
- Call for transesophageal echocardiography
- Check pulse. If no pulse:
  - Start chest compressions
  - Give epinephrine 1 mg
  - If cardiac arrest, See CHKLIST 4 (Arrest)
  - Consider ECMO (pager #2871)

Air Embolus ADULT

Rev. Aug 25, 2016
Human Factors Design B. Burian et al

2 Anaphylaxis ADULT

VERIFY DX – STABILIZE PATIENT

- Verify Anaphylaxis, may have: Tachycardia, hypotension, rash, bronchospasm
- Give 100% O_2, evaluate ventilation
- Remove suspected trigger(s)
  - If latex is suspected, thoroughly wash area, have surgeons change to non-latex gloves
  - If HYPOtensive, turn off anesthetic agents

TREATMENT

Cause	Action
Decreased intravascular volume	NS or LR 1 L IV *rapidly*, may need 5-10 L
Hypotension Bronchospasm	Epinephrine 10-100 mcg IV/IO, as needed, may need infusion 0.02-0.2 MICROgrams/kg/min
Hypotension refractory to epinephrine	Vasopressin 1-2 Units IV
Bronchospasm	Albuterol 4-10 puffs or more as needed
Mediator release	Hydrocortisone 100 mg IV
Histamine release	Diphenhydramine 50 mg IV *and* famotidine 20 mg IV
**Partial Differential**	
Fat, thrombotic, cement, amniotic fluid embolus	See CHKLST 1 (embolus)
Sepsis	Support BP, antibiotics
Myocardial Ischemia	Morphine, oxygen, aspirin, NTG, beta-blocker, heparin

DRUG / DOSAGE SUMMARY

- **Epinephrine** 10-100 mcg IV, may need infusion 0.02-0.2 MICROgrams/kg/min IV
- **Vasopressin** 1-2 units IV
- **Albuterol** 4-10 puffs or more if needed
- **Hydrocortisone 100** mg IV
- **Diphenhydramine** 50 mg IV
- **Famotidine** 20 mg IV

CRISIS MANAGEMENT (If Severe)

- Notify surgeon, call for help and code cart
- Check pulse. If no pulse:
  - Start chest compressions
  - Give epinephrine 1 mg IV/IO
  - If cardiac arrest, See CHKLIST 4 (Arrest)
  - Consider ECMO (pager #2871)

Common CAUSATIVE Agents

- Neuromuscular blockers
- Antibiotics
- Latex
- Chlorhexidine prep solution
- IV colloids

3 Bradycardia/Pacing ADULT

VERIFY DX – STABILIZE PATIENT

- Verify Bradycardia: ↓ HR ↓ BP with clinical evidence of poor perfusion
- Give 100% O_2, evaluate ventilation
- Consider
  - Glycopyrrolate 0.2-0.4 mg IV *or*
  - Atropine 0.1-0.4 mg IV

TREATMENT

Cause	Action
Surgical stimulation	▪ If laparoscopic, de-sufflate<br>▪ If not laparoscopic, stop surgery
Beta-blocker overdose	▪ Glucagon 3-10 mg IV, then 0.07 mg/kg/hour IV infusion
Ca-channel blocker overdose	▪ Calcium chloride 1 mg IV *or*<br>▪ Calcium gluconate 1-2 mg IV<br>▪ If ineffective, glucagon 3-10 mg IV, then 0.07 mg/kg/hour IV infusion
High spinal	▪ Intubate, ventilate, support HR and BP
External/internal pacer failure	▪ Contact electrophysiology (pager #4118)
Cardiac arrest	▪ See CHKLST 4 (Arrest)
Myocardial ischemia	▪ O_2, NTG, morphine, beta-blockers (when tolerated), consider risks/benefits of aspirin, clopidrogel if acute coronary syndrome

DRUG / DOSAGE SUMMARY

- **Atropine** 0.1-0.4 mg IV
- **Calcium chloride** 1 mg IV
- **Calcium gluconate** 1-2 mg IV
- **Epinephrine** 50 mcg-1 mg IV
- **Glucagon** 3-10 mg IV, *then* 0.07 mg/kg/hour IV infusion
- **Glycopyrrolate** 0.2-0.4 mgIV

CRISIS MANAGEMENT (If Severe)

- Notify surgeon, call for help and code cart
- Check pulse. If no pulse:
  - Start chest compressions
  - Give epinephrine 1 mg IV
  - Consider transcutaneous pacing (see inset)
  - If cardiac arrest, See CHKLIST 4 (Arrest)
  - Consider ECMO (pager #2871)

Instructions for PACING

1. Call for Pacer/Defibrillator
2. Place pacing ECG electrodes *and* pacer pads on chest.
3. Turn monitor/defibrillator ON, set to PACER mode
4. Set PACER RATE (ppm) to desired rate/min (80).

   Can be adjusted up or down based on clinical response once pacing is established
5. Increase the PACER OUTPUT (mA) until electrical capture

   Pacer spikes aligned with QRS complex. Threshold normally 65-100mA
6. Set PACER OUTPUT to 10mA above this level
7. Confirm pulse is present. If not, repeat steps 4-5

Rev Aug 25, 2016
Human Factors Design
B. Burian et al

4 Cardiac Arrest ADULT

VERIFY DX – STABILIZE PATIENT

- If no pulse: start chest compressions
- Give 100% O_2. Turn off all anesthetic gases and anesthetic infusions

REVERSIBLE CAUSES: Hs and Ts

- Hypovolemia
- Hypoxemia
- Hydrogen ion (acidosis)
- Hyperkalemia (see CHKLST 5)
- Hypoglycemia
- Hypothermia

- Tension Pneumothorax (see CHKLST 10)
- Tamponade, cardiac
- Tamponade, pulmonary (breath stacking or excessive auto-PEEP)
- Thrombosis (if air embolus, see CHKLST 1)
- Toxin (anesthetic, β-blocker. If LAST, see CHKLST 7)
- Trauma (see CHKLST 9, massive hemorrhage)

DRUG / DOSAGE SUMMARY

- **Epinephrine** 1 mg IV q 3-5 min
- **Amiodarone** first dose 300 mg IV, second dose 150 mg IV

CRISIS MANAGEMENT

- Notify surgeon, call for help and code cart/defibrillator
  - Attending: announce 'I am the team leader'
    - Say: 'The #1 priority is high quality chest compressions'
- Continue chest compressions (100/min + 8 breaths/min)
  - Maximize $EtCO_2$ > 10 mmHg and if art line, DBP > 20 mmHg with force/depth of compressions
  - Allow full chest recoil between compressions
  - Switch with another provider every 2 min, if possible
  - Use sudden increase in $EtCO_2$ for ROSC. Do not stop compressions for pulse check
- Attach defibrillator pads
- Check pulse and rhythm every 2 minutes during compressor switch
- IF VF/VT, shock at 150 J (all DACC defibrillators are biphasic)
- **Immediately** resume compressions
- Give epinephrine 1 mg IV q 3-5 min
- Consider ETT if not already in place
- If VF/VT, give amiodarone 300 mg IV (2nd dose: 150 mg IV)
- Check for reversible causes (Hs and Ts) early and often (see orange box)
- If cardiac arrest >6 min, activate ECMO (pager #2871)

5 Hyperkalemia ADULT

VERIFY DX – STABILIZE PATIENT

- Verify Hyperkalemia. May have: K+>6, ECG changes (see orange box)
- Hyperventilate, give 100% O_2, evaluate ventilation
- Give calcium gluconate 1-2 g IV or calcium chloride 1 g IV
  - If peripheral IV, visually confirm no infiltration while injecting
  - Flush tubing after calcium administration

TREATMENT

Cause	Action
Infusion of K+	▪ Stop infusion of PRBCs/LR, switch to NS
Increased K+ in blood	▪ Calcium gluconate/chloride 1-2 g IV<br>▪ Sodium bicarbonate 100 mEq IV<br>▪ Albuterol 4-10 puffs through ETT or nebulized (10-20 mg in 4 mL saline)<br>▪ Dextrose 25-50 g IV *and* Insulin 10 Units IV<br>▪ Furosemide 20-40 mg IV<br>▪ Terbutaline 0.25 mg loading dose *subcutaneously* then 0.1-10 MICROgrams/kg/min IV<br>▪ Kalexate 15 g OG/NG
Refractory hyperkalemia	▪ Dialysis
Ongoing transfusion need	▪ Use washed or fresh PRBCs

Partial Differential

Embolus: Fat, thrombotic, cement, amniotic fluid	▪ See CHKLST 1 (embolus)
Anaphylaxis	▪ See CHKLIST 2 (anaphylaxis)
Local Anesthetic Systemic Toxicity	▪ See CHKLIST 8 (LAST)

DRUG / DOSAGE SUMMARY

- **Calcium gluconate or chloride** 1-2 g IV
- **Sodium bicarbonate** 100 mEq IV
- **Albuterol** 4-10 puffs through ETT or nebulized (10-20 mg in 4 mL saline)
- **Dextrose** 25-50 g IV and **Insulin** IV 10 Units IV
- **Furosemide** IV 20-40 mg IV
- **Terbutaline** 0.25 mg loading dose *subcutaneously* then 0.1-10 MICROgrams/kg/min IV
- **Kalexate** 15 g OG/NG

CRISIS MANAGEMENT (If Severe)

- Notify surgeon, call for help and code cart
- Check pulse. If no pulse:
  - Start chest compressions
  - Give epinephrine 1 mg IV
  - If cardiac arrest, See CHKLST 4 (Arrest)
  - Consider ECMO (pager #2871)

ECG Manifestations

- Tall peaked T waves
- Prolonged PR
- Wide QRS
- Heart block
- Sine wave
- V fib or asystole

Human Factors Design B. Burian et al
Rev Aug 25, 2016

6 Increased Intracranial Pressure ADULT

VERIFY DX – STABILIZE PATIENT

- Verify Increased ICP: above normal value of 8-12 mmHg
  CPP = MAP - ICP
- If GCS < 8, respiratory distress, or hemodynamic instability:
  - Secure airway
  - Provide sedation prior to transport
  - Keep $PaCO_2$ 30-35 mmHg and PaO_2 > 60 mmHg

DRUG / DOSAGE SUMMARY

- **Hypertonic saline 3%** (via central line) 1-3 mL/kg IV over 20 min, then 0.1-1 mL/kg/hour IV
- **Mannitol** 0.25-1 g/kg IV
- **Keppra** (levetiracetam) 10 mg/kg IV

TREATMENT

Cause		Action
Increased intracranial contents	Blood	▪ HOB at 30-45° and neck neutral with 2 fingerbreadths between neck and chest to maintain venous outflow<br>▪ TIVA for anesthesia maintenance +/- 0.5 MAC inhaled agent<br>▪ Avoid high peak airway pressures
	Brain	▪ Hypertonic saline 3% (via central line) 1-3 mL/kg IV over 20 min, then 0.1-1 mL/kg/hour IV. When in use, monitor serum sodium, keep osmolarity <360 mOsm/L<br>▪ Mannitol 0.25-1 g/kg IV<br>▪ Decadron 10 mg IV or equivalent
	CSF	▪ Furosemide 10-20 mg<br>▪ Ask surgeon to drain CSF if feasible
Refractory elevated ICP		▪ Consider hypothermia - temperature 34°C. (Weak evidence and may impair neurologic exam; consult with care team)<br>▪ Neuromuscular blockade with a nondepolarizing drug

Need	Action
Increase MAP to maintain CPP >50	▪ Phenylephrine 0.1 mg IV and/or 0.1- 0.5 mcg/kg/min IV<br>▪ Norepinephrine 0.05-2 MICROgrams/kg/min IV
Seizure prophylaxis	▪ Keppra (levetiracetam) 10 mg/kg IV
Brain protection	▪ Avoid hyperthermia<br>▪ Avoid hyperglcemia & dextrose containing solutions (maintain glucose level < 200 mg/dL)

7 Local Anesthetic Systemic Toxicity ADULT

VERIFY DX – STABILIZE PATIENT

- Verify Local Anesthetic Systemic Toxicity (LAST), may have:
  - CNS symptoms (may not occur): tinnitus, metallic taste, seizures
  - CV collapse: rhythm disturbance, altered consciousness
- Give 100% O_2, evaluate ventilation, stop N_2O and volatiles
- Stop local anesthetic
  - What was given? Drug, dose, site? Does this = a crisis or not?
  - If crisis, **request intralipid** (available in CCD block cart, DCAM block room, CLI carts, pharmacy. Map, CHKLST 12-15)
- Ensure adequate IV access, monitoring of continuous ECG, BP, and SaO_2
- Avoid propofol, vasopressin, calcium channel blockers

TREATMENT

Cause/Need	Action
Need for seizure suppression	▪ Midazolam 2-8 mg IV
Hypotension	▪ Epinephrine 10-100 mcg IV/IO, as needed, may need infusion 0.02-0.2 MICROgrams/kg/min
Arrhythmias	▪ Amiodarone 150 mg IV over 10 min
Acidosis, hyperkalemia	▪ Draw serial ABGs, consider a-line<br>▪ Hyperkalemia tx, see CHKLIST 5
**Partial Differential**	
Embolus: Air, fat, thrombotic, cement, amniotic fluid	▪ See CHKLST 1 (air embolus)
Anaphylaxis	▪ See CHKLST 2 (anaphylaxis)
Myocardial Ischemia	▪ Morphine, aspirin, NTG, beta-blocker, heparin

DRUG / DOSAGE SUMMARY

- **Intralipid 20%** 100 mL IV over 1 min, repeat until stable (see orange box below)
- **Epinephrine** 10-100 mcg IV/IO
- **Midazolam** 2-8 mg IV
- **Amiodarone** 150 mg IV over 10 min

CRISIS MANAGEMENT (If Severe)

- Notify surgeon, call for help and code cart
- Check pulse. If cardiac instability occurs:
  - Start chest compressions (lipid must circulate)
  - Give epinephrine 10-100 MICROgrams/dose
  - If cardiac arrest, See CHKLIST 4 (Arrest)
  - Consider ECMO (pager #2871)

Intralipid Dosing

- Bolus **Intralipid 20% 100 mL IV** over 1 minute (~1.5 mL/kg lean body weight). Repeat every 3-5 minutes, until hemodynamically stable
  - Start infusion **0.25 mL/kg/min**, increase as needed to 0.5 mL/kg/min
- **Continue infusion for at least 10 min** after hemodynamic stability is restored.
- Max dose of intralipid 20% is 10 mL/kg (3 250 mL bags) over first 30 min. If not responding, reconsider if other diagnosis or inadequate resuscitation

8 Malignant Hyperthermia ADULT

MH hotline
1-800-644-9737

VERIFY DX – STABILIZE PATIENT

- Verify MH, may have: ↑ CO_2, ↑ HR, ↑ Temp
- **Notify surgeon, call for help, call for MH cart** (CCD: in core next to OR 18, Comer: in anesthesia workroom)
- Stop procedure, if possible
- Stop volatile and succinylcholine and transition to non-triggering anesthetic
- **Give dantrolene** 2.5 mg/kg IV every 5 min until symptoms resolve
  - Dantrium/Revonto: Assign dedicated person to mix these formulations of dantrolene (20 mg/vial) with 60 mL non-bacteriostatic sterile water (on MH cart)
  - Ryanodex: 250 mg is mixed with 5 mL non-bacteriostatic sterile water

DRUG / DOSAGE SUMMARY

- **Dantrolene** 2.5 mg/kg IV, repeat q5 min if sx
- **Sodium bicarbonate** 1-2 mEq/kg IV
- **Calcium gluconate/chloride** 1-2 g IV
- **Insulin** 10 units and **dextrose** 25-50 g

CRISIS MANAGEMENT (If Severe)

- Notify surgeon, call for help and code cart
- Check Pulse. If no pulse:
  - Start chest compressions
  - Give epinephrine 1 mg IV/IO
  - If cardiac arrest, See CHKLIST 4 (Arrest)
  - Consider ECMO (pager #2871)

TREATMENT

- Place an a-line and additional IVs, if needed
- Get serial ABGs or VBGs, electrolytes, serum CK, serum/urine myoglobin, coagulation
- Place urinary catheter to monitor urine output for goal >1mL/kg/hour

IF	Action
Volatile in circuit	▪ Attach charcoal filters to inspiratory and expiratory limbs - replace hourly in a patient exhaling volatile anesthetics
Acidosis	▪ Hyperventilate with FiO2 100% flow >10 L/min<br>▪ Give sodium bicarbonate 1-2 mEq/kg IV for suspected metabolic acidosis; maintain pH > 7.2
Temperature > 39° C	▪ Apply ice externally to axilla, groin and around head<br>▪ Change IV fluids to cold saline<br>▪ Lavage body cavities (NG, foley) with cold water<br>▪ Stop cooling when temperature < 38° C
Hyperkalemia	▪ Calcium gluconate 1-2 g IV or calcium chloride 1 g IV; If peripheral IV, visually confirm no infiltration while injecting<br>▪ Regular insulin 10 units and dextrose 25-50g
Dysrhythmias	▪ Do *not* use calcium channel blocker; standard antiarrhythmics are acceptable

9 Massive Hemorrhage: Transfusion ADULT

VERIFY DX – STABILIZE PATIENT

- Verify massive hemorrhage, may have: ongoing and/or severe bleeding
- Give 100% O_2, evaluate ventilation
- Notify blood bank immediately (x26827), **request massive transfusion protocol**. Blood boxes will keep coming
  - Box contents: PRBCs 6, FFP 4, Platelet 6 units (1 pooled pack)

TREATMENT

IF	Action
Ongoing bleeding	▪ Begin transfusion when available<br>▪ Consider RBC:FFP:Platelets 2:1:1: or 1:1:1<br>▪ Consider intraoperative blood salvage (Cell Saver)<br>▪ Obtain additional vascular access<br>▪ Warm the room
Crossmatched blood not available yet	▪ Initially use un-crossed matched O negative blood
Need to monitor bleeding/coagulation/ electrolytes	▪ Send labs q30min<br>• CBC, platelets, PT/PTT/INR, fibrinogen<br>• ABG, Na, K, Ca, lactate
Refractory hemorrhage	▪ Consider rFactor VIIa
**Suspected hyperkalemia**	▪ Give calcium gluconate 1-2 g IV or calcium chloride 1 g IV; If peripheral IV, visually confirm no infiltration while injecting
Bleeding is controlled	▪ Call blood bank to terminate massive transfusion protocol

CRISIS MANAGEMENT (If Severe)

- Notify surgeon, call for help and code cart
- Give calcium gluconate 1-2 g IV or calcium chloride 1 g IV; If peripheral IV, visually confirm no infiltration while injecting
- Check pulse. If no pulse:
  - Start chest compressions
  - Give epinephrine 1 mg IV/IO
  - If cardiac arrest, See CHKLIST 4 (Arrest)
  - Consider ECMO (pager #2871)

Blood Product Administration

- Use 140 micron filter for all products
- Use a blood warmer for RBC and FFP transfusion (NOT for platelets)
- Rapid transfusion pumps may be used when increased flow is needed

10 Tension Pneumothorax ADULT

VERIFY DX – STABILIZE PATIENT

- Verify Anaphylaxis, may have: ↑ HR, ↓ SpO_2 ↓ BP, tracheal deviation, mediastinal shift
- Give 100% O_2, evaluate ventilation, stop N_2O
- Secure airway with endotracheal tube
- Reduce positive ventilation pressure
- Perform immediate needle decompression, then chest tube placement (see 'treatment')

DRUG / DOSAGE SUMMARY

- **Epinephrine** 10 MICROgrams - 1 mg or infusion 0.02-1 MICROgrams/kg/min
- **Norepinephrine** 0.05-2 MICROgrams/kg/min IV

CRISIS MANAGEMENT (If Severe)

- Notify surgeon, call for help and code cart
- Check pulse. If no pulse:
  - Start chest compressions
  - Give epinephrine 1 mg IV/IO
  - If cardiac arrest, See CHKLIST 4 (Arrest)
  - Consider ECMO (pager #2871)

TREATMENT

IF	Action
Collapsed lung	▪ Needle decompression: 2nd rib space superior to 3rd rib, mid-clavicular line (14-16g IV catheter)<br>▪ Chest tube insertion (5-6th intercostal space, mid-axillary line)
Circulatory collapse	▪ **Epinephrine** 10 MICROgrams-1 mg or infusion 0.02-1 MICROgrams/kg/min<br>▪ **Norepinephrine** 0.05-2 MICROgrams/kg/min IV
No improvement in BP after needle decompression	▪ Consider<br>• Needle decompression of contralateral side<br>• Presence of pneumopericardium

Partial Differential

Cause	Action
Auto-PEEP	▪ Increase expiratory time
Severe bronchospasm	▪ Albuterol (4-10 puffs or more) through ETT<br>▪ Epinephrine 10-100 MICROgrams IV<br>▪ Ketamine 10-200 mg IV
Anaphylaxis	▪ See CHKLST 2 (anaphylaxis)
Air Embolus	▪ See CHKLST 1 (air embolus)

11 Transfusion Reactions ADULT

STABILIZE PATIENT

- Give 100% O_2, evaluate ventilation
- Stop transfusion, disconnect donor product and get new IV tubing
- Examine blood product ID; determine if correct patient
- Send product to blood bank (x26827)
- Verify Transfusion Reaction, must determine type (see green box below)

DRUG / DOSAGE SUMMARY

- **Furosemide** 0.1 mg/kg (for hemolitic)
- **Mannitol** 0.5 g/kg (for hemolitic)
- **Dopamine** 2-4 MICROgrams/kg/min IV (for hemolitic)
- **Epinephrine** 10 -100 MICROgrams IV (for anaphylactic)
- **Diphenhydramine** 25-50 mg IV (for anaphylactic)
- **Hydrocortisone** 50-100 mg IV (for anaphylactic)

CRISIS MANAGEMENT (If Severe)

- Notify surgeon, call for help and code cart
- Check pulse. If no pulse:
  - Start chest compressions
  - Give epinephrine 1 mg IV/IO
  - If cardiac arrest, See CHKLIST 4 (Arrest)
  - Consider ECMO (pager #2871)

**VERIFY DX**	**Hemolytic:**<br>Hemoglobinemia, hemoglobinuria, DIC, ↓ BP, ↑ HR, bronchospasm	**Non-Hemolytic:**<br>↓ BP, bronchospasm, pulmonary edema, fever, rash	**Anaphylactic:**<br>Erythema, urticaria, angioedema, bronchospasm, ↑ HR, shock
**TREATMENT**	▪ Furosemide 0.1 mg/kg<br>▪ Mannitol 0.5 g/kg<br>▪ Dopamine 2-4 MICROgrams/kg/min<br>▪ Maintain urine output at least 1-2 mL/kg/hr<br>▪ Prepare for cardiovascular instability<br>▪ Send blood and urine sample to laboratory	▪ Treat fever<br>▪ Treat pulmonary edema<br>▪ Observe for signs of hemolysis	▪ Epinephrine 10-100 MICROgrams IV<br>▪ Diphenhydramine 25-50 mg IV<br>▪ Hydrocortisone 50-100 mg IV<br>▪ Maintain intravascular volume

**TREATMENT (cont.)**<br><br>**Partial Differential**	**Cause**	**Action**
	Embolus: Air, Fat, thrombotic, cement, amniotic fluid	▪ See CHKLST 1 (air embolus)
	Anaphylaxis	▪ See CHKLST 2 (anaphylaxis)
	LAST	▪ See CHKLST 7 (LAST)
	Cardiac Ischemia	▪ Morphine, oxygen, aspirin, NTG, beta-blockers, heparin

Revision July 26, 2016
Human Factors Design
B. Burian et al
