# Supplementary material for: Why are critical event checklists not always used in the perioperative setting?: A retrospective survey
Source: PLoS One. 2025 Feb 28;20(2):e0314774. doi: 10.1371/journal.pone.0314774 (PMC11870359; doi:10.1371/journal.pone.0314774)
Supplement: S3 File — (PDF) [file pone.0314774.s003.pdf]

Critical Event Cognitive Aids: Why, How, and Implementation (here at DACC!)

Anna Clebone, M.D.

THE UNIVERSITY OF CHICAGO MEDICINE

- LEARNING OBJECTIVES
- How to create cognitive aids for the medical environment
  - What NOT to do (we are not pilots in the OR)
  - What TO do (adapt the cognitive aid to us, don’t try to make us more like pilots)
  - How this could work at DACC

THE UNIVERSITY OF CHICAGO MEDICINE

- What is a cognitive aid?

ELECTRICAL FIRE IN FLIGHT

Master Switch . . . . OFF
All Other Switches Except Ignition . . . . OFF
Vents Heat / Air . . . . CLOSED
Fire Extinguisher . . . . ACTIVATE

WARNING: AFTER DISCHARGING FIRE EXTINGUISHER WITHIN CLOSED CABIN, VENTILATE CABIN

IF FIRE APPEARS OUT AND ELECTRICAL POWER IS NECESSARY FOR CONTINUANCE OF FLIGHT

Master Switch . . . . ON
Circuit Breakers . . . . check for faulty circuit, do not reset
Radio / Electrical Switches . . . . ON
(One at a time, with delay after each until short circuit is localized.)
Vents Heat / Air . . . . OPEN
(When it is ascertained that fire is completely extinguished.)

Source: Cessna 172 handbook

Marshall, S. The use of cognitive aids during emergencies in anesthesia: a review of the literature. Anesth Analg. 2013 Nov;117(5):1162-71.

Clebone A, Burian B, Watkins S, Galvez J, Lockman J, Heitmiller C, SPA Q and S Committee, The Development and Implementation of Cognitive Aids for Critical Events in Pediatric Anesthesia: Submitted for Publication

THE UNIVERSITY OF CHICAGO MEDICINE

- Why have critical events cognitive aids?
    1) Repository of latest evidence-based and expert-opinion based information
    2) Source of just in time information
    3) Method to facilitate a shared understanding
    4) Guide to local resources

Marshall, S. The use of cognitive aids during emergencies in anesthesia: a review of the literature. Anesth Analg. 2013 Nov;117(5):1162-71.

Clebone A, Burian B, Watkins S, Galvez J, Lockman J, Heitmiller C, SPA Q and S Committee, The Development and Implementation of Cognitive Aids for Critical Events in Pediatric Anesthesia: Submitted for Publication

THE UNIVERSITY OF CHICAGO MEDICINE

What is a perioperative critical event?

- An unexpected, infrequent, potentially harmful event, for which multiple correct actions must be quickly and correctly performed to minimize the risk of patient injury.

NOT a critical event	A critical event
Known latex allergy	**Anaphylaxis**
Chronic angina and ST changes	**Acute Myocardial Ischemia**
Fever in a septic patient	**Malignant Hyperthermia**

- Other critical events: Air Embolus, Bradycardia/ Pacing, Cardi[illegible] Hemorrhage

THE UNIVERSITY OF CHICAGO MEDICINE

Why are critical events cognitive aids used?

- Contains the steps that are needed to respond to a specific time critical event. E.g. hyperkalemia
- "Series of tasks is too long to be committed to memory (or there are likely to be interruptions to execution of the task that might interfere with memory retrieval)"
- Used for situations in which there is an advantage to taking specific steps

Clay-Williams R[1], Colligan L[2]. **Back to basics: checklists in aviation and healthcare.** BMJ Qual Saf. 2015 Jul;24(7):428-31. doi: 10.1136/bmjqs-2015-003957. Epub 2015 May 12.

- Why do we need cognitive aids:
  - Performance goes down under pressure
  - Rare situations
  - Common situations where our initial solutions don't work
  - Tailored to our local resources and easily accessible
    - Guide to getting more resources (ECMO pager number)
    - Infrequently used equipment (pacer)

THE UNIVERSITY OF CHICAGO MEDICINE

- Performance goes down under pressure

Burian BK. Factors affecting the use of emergency and abnormal checklists: Implications for current and NextGen operations. NASA Technical Memorandum TM-2014-218382. Moffett Field, CA: NASA Ames Research Center, 2014.

Stiegler MP, Neelankavil JP, Canales C, Dhillon A. Cognitive errors detected in anaesthesiology: a literature review and pilot study. Br J Anaesth 2012;108:229-35.

Sebbag I1, Frequency of intraoperative cardiac arrest and medium-term survival. Sao Paulo Med J. 2013;131(5):309-14.

Andersen LW, Berg KM, Saindon BZ, Massaro JM, Raymond TT, Berg RA, Nadkarni VM, Donnino MW. Time to Epinephrine and Survival After Pediatric In-Hospital Cardiac Arrest. Jama 2015;314:802-10.

Howard-Quijano KJ, Stiegler MA, Huang YM, Canales C, Steadman RH. Anesthesiology residents' performance of pediatric resuscitation during a simulated hyperkalemic cardiac arrest. Anesthesiology 2010;112:993-7.

Neumar RW, Otto CW, Link MS, Kronick SL, Shuster M, Callaway CW, Kudenchuk PJ, Ornato JP, McNally B, Silvers SM, Passman RS, White RD, Hess EP, Tang W, Davis D, Sinz E, Morrison LJ. Part 8: adult advanced cardiovascular life support: 2010 American Heart Association Guidelines for Cardiopulmonary Resuscitation and Emergency Cardiovascular Care. Circulation 2010;122:S729-67.

THE UNIVERSITY OF CHICAGO MEDICINE

- Rare Situations
  - MH: Mortality decreases from 70-80% to <5% with early treatment
  - Wide acceptance of MH algorithm

Dong-Chan Kim. Malignant hyperthermia. Korean J Anesthesiol. 2012 Nov; 63(5): 391–401.

THE UNIVERSITY OF CHICAGO MEDICINE

- Tailored to our local resources and easily accessible
  - Bing (default web browser here at DACC) search for MH
  - Screen also needed for EMR

THE UNIVERSITY OF CHICAGO MEDICINE

mh treatment

Back to Bing search

Sign in 1

Web Images Videos Maps News Explore

3,940,000 RESULTS Any time

Mental Health Treatment & Services - NAMI: The National ...
www.nami.org › Learn More
Learn about the different **treatments**, supports and services used to **treat mental health**.

[PDF] **ACUTE PHASE TREATMENT - MH** Hotline Information
medical.**mhaus**.org/PubData/PDFs/treatmentposter.pdf
malignant hyperthermia mh hotline 1-800-644-9737 outside the us: 1-315-464-7079 diagnosis vs. associated problems **acute phase treatment** post acute phase signs of **mh**:

Mental Health Treatments | **Mental Health** America
www.mentalhealthamerica.net › Finding Help
Psychotherapy – Psychotherapy is the therapeutic **treatment** of mental illness provided by a trained **mental health** professional. Psychotherapy explores thoughts ...

SAMHSA Behavioral Health **Treatment** Services Locator
https://www.find**treatment**.samhsa.gov
Behavioral Health **Treatment** Services Locator. Welcome to the Behavioral Health **Treatment** Services Locator, a confidential and anonymous source of information for ...

National Institute of Mental Health - Official Site
www.nimh.nih.gov
The **National Institute of Mental Health** (NIMH) is the largest scientific organization in the world dedicated to research focused on the understanding, **treatment**, and ...

View the **MH Treatment** Guidelines - **MHAUS**
www.**mhaus**.org/healthcare-professionals/managing-a-crisis
Emergency **Treatment** for An Acute **MH** Event. The following four things should be done as soon as possible: Notify surgeon to halt the procedure ASAP: Discontinue ...

New York State **Office of Mental Health**
www.omh.ny.gov
New York State **Office of Mental Health** ... OnTrackNY is an innovative **treatment** program operating in 10 locations across New York for adolescents and ...

Malignant Hyperthermia Acute **Treatment**: **MH** Investigation ...
pie.med.utoronto.ca/**MH**/**MH**_content/acute**Treatment**.html
Acute **treatment** of **MH** - MHIU, **malignant hyperthermia**, anesthesia, medicine, **MH**, Dantrolene, caffeine-halothane contracture test, CHCT

Home | **MentalHealth**.gov
https://www.mentalhealth.gov
MentalHealth.gov provides one-stop access to U.S. government **mental health** information. This site explains the basics of **mental health**, myths and facts, **treatment** ...

Treatments for Mental Disorders | SAMHSA
www.samhsa.gov › ... › Behavioral Health **Treatments** and Services
Treatments for Mental Disorders. ... Also included are **data** on the use of **mental health** and substance use **treatment** services by Medicare enrollees, ...

Related searches for **mh treatment**
- Malignant Hyperthermia Code Sheet
- Malignant Hyperthermia Protocol 2013
- Malignant Hyperthermia Poster 2015
- Malignant Hyperthermia PowerPoint for Nurses
- Malignant Hyperthermia **Treatment**
- Malignant Hyperthermia Record Sheet

Related searches
- Malignant Hyperthermia Code Sheet
- Malignant Hyperthermia Record Sheet
- Malignant Hyperthermia Poster 2015
- Malignant Hyperthermia Protocol PDF
- Malignant Hyperthermia Protocol 2013
- Malignant Hyperthermia PowerPoint for Nurses
- Malignant Hyperthermia **Treatment**
- Montgomery County **MH Treatment** Facility

Schizophrenia **Treatment** | Hanbleceya.com
Ad · www.Hanbleceya.com
Treating Schizophrenia Since 1979. Intensive **Therapy**, Housing & Support
Mental Health Treatment Center

Mental Health Treatment | RoseHillCenter.org
Ad · www.RoseHillCenter.org
Residential Center Helping Adults Overcome **Mental** Illness.

See your ad here »

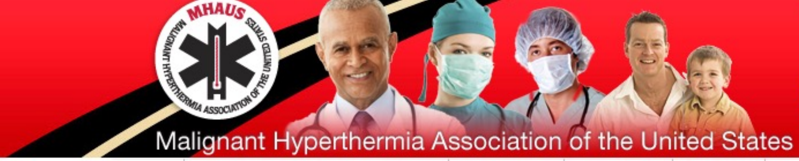

Managing An MH Crisis

How To Be Prepared
Managing an MH Crisis
After an MH Crisis
Post-MH Event Plan and Counseling
MHAUS Recommendations
Professional Development
Frequently Asked Questions
MHAUS Website Directory

Emergency Treatment for An Acute MH Event

The following four things should be done as soon as possible:

- Notify surgeon to halt the procedure ASAP: Discontinue volatile agents and succinylcholine.

– If surgery must be continued, maintain general anesthesia with IV non-triggering anesthetics (e.g., IV sedatives, narcotics, amnestics and non-depolarizing neuromuscular blockers as needed)

- Get dantrolene/MH cart. (Call 911 if surgicenter)

– Call for help within your institution; also, call the MHAUS Hotline (1-800-644-9737) for additional advice. (Outside the US, please call: 001-209-417-3722)

- Hyperventilate with 100% oxygen at flows of 10L/min to flush volatile anesthetics and lower ETCO2. If available, insert activated charcoal filters (Vapor-Clean™, Dynasthetics, Salt Lake City, UT) into the inspiratory and expiratory limbs of the breathing circuit. The Vapor-Clean™ filter may become saturated after one hour; therefore, a replacement set of filters should be substituted after each hour of use.
- Give IV dantrolene 2.5 mg/kg rapidly through large-bore IV, if possible. Repeat as frequently as needed until the patient responds with a decrease in $ETCO_2$, decreased muscle rigidity, and/or lowered heart rate. Large doses (>10mg/kg) may be required for patients with persistent contractures or rigidity.

– DANTRIUM®/REVONTO® – Each 20 mg vial should be reconstituted by adding 60 ml of sterile water for injection, USP (without a bacteriostatic agent) and the vial shaken until the solution is clear.

– RYANODEX®– Each 250 mg vial should be reconstituted with 5 ml of sterile water for injection, USP (without a bacteriostatic agent) and shaken to ensure an orange-colored uniform, opaque suspension.

If giving large doses (> 10 mg/kg) without symptom resolution, consider alternative diagnoses.

Obtain blood gas (venous or arterial) to determine degree of metabolic acidosis. Consider administration of sodium bicarbonate, 1-2 mEq/kg dose, for base excess greater than -8 (maximum dose 50 mEq).

Cool the patient if core temperature is >39°C or less if rapidly rising. Stop cooling when the temperature has decreased to <38°C.

If hyperkalemia (K > 5.9 or less with ECG changes) is present, treat with:

- Calcium chloride 10 mg/kg (maximum dose 2,000 mg) or calcium gluconate 30 mg/kg (maximum dose 3,000 mg) for life-threatening hyperkalemia
- Sodium bicarbonate

Donate
Support MHAUS Today!
Membership
Join MHAUS Today!
MH Registry
Support MH Research, Join Today!

Ryanodex® (dantrolene sodium) for injectable suspension
Learn more
RYA-MH-2016-038

Click Here for Information about Dantrium® IV (dantrolene sodium for injection)
PAR STERILE PRODUCTS PSPB03 0515

MHAUS Membership Campaign
Refer members, Get Rewards!

Feed MH Research!
Get the story.
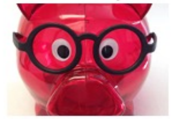
Click here!

Shop at AmazonSmile
and Amazon will make a donation to:
MHAUS
Get started

- Simple to use, but not simple to engineer . … .

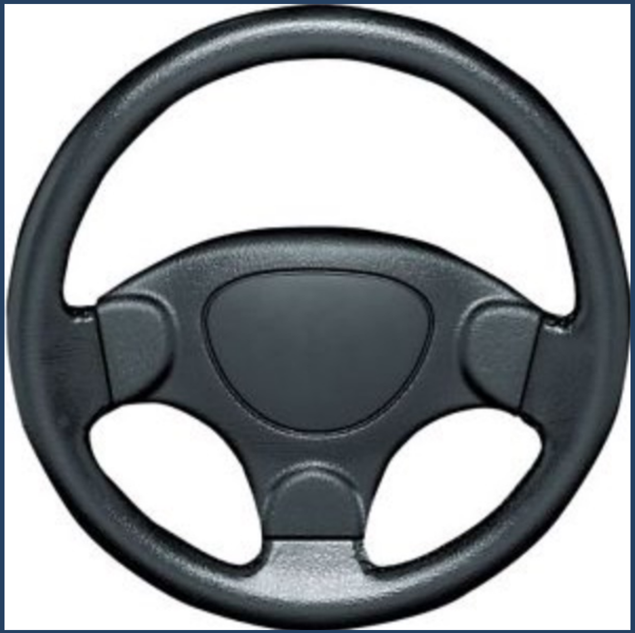

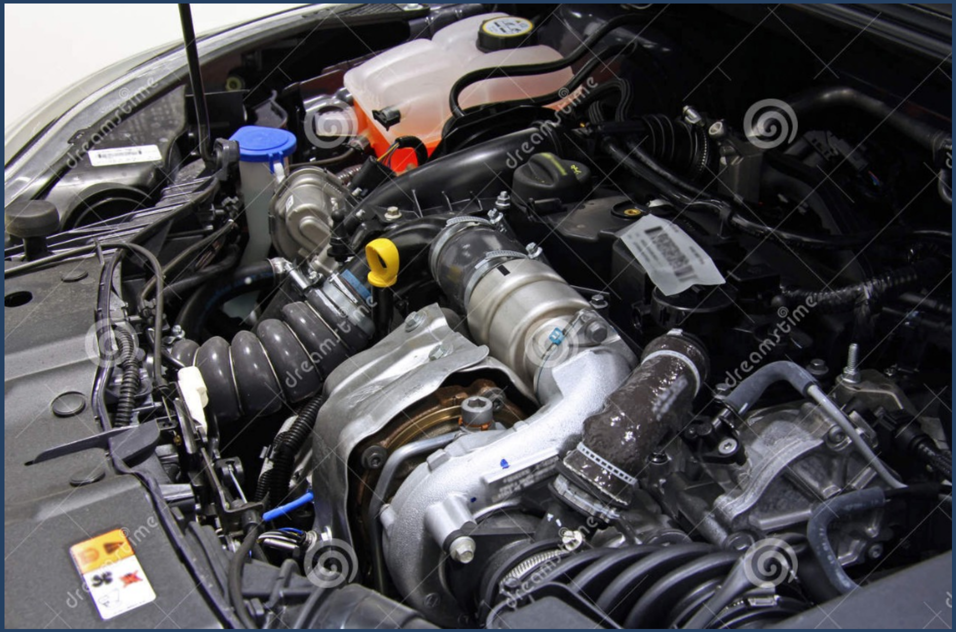

THE UNIVERSITY OF CHICAGO MEDICINE

What Doesn't Work?

How to fix: ask yourself

1) Aid doesn't match purpose and environment

1) Why needed and what change do we want?

2) Bad content and poor design

2) Content serves purpose? Does design facilitate, not hinder?

0 Does

3) cultural barriers: professional, institutional

3) Clear purpose? Did you invite members of all potential user groups to create and implement?

4) insufficient or non-existent induction and/or training

4) What training is needed?

Burian, et al, submitted for publication

What Doesn't Work?

1) Aid doesn't match purpose and environment

2) Bad content and poor design

3) cultural barriers: professional, institutional

4) insufficient or non-existent induction and/or training

How to fix: ask yourself

1) Why needed and what change do we want?

2) Content serves purpose? Does design facilitate, not hinder?

0 Does

3) Clear purpose? Did you invite members of all potential user groups to create and implement?

4) What training is needed?

Burian, et al, submitted for publication

Aid doesn't match setting and purpose

Checklist and Setting Mismatch

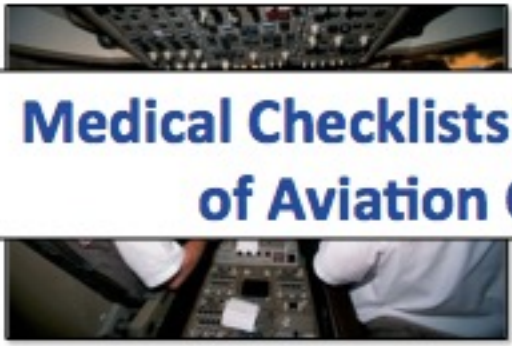

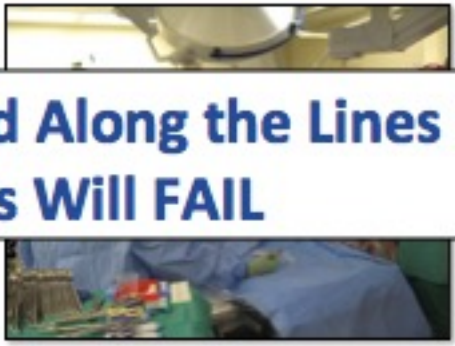

Medical Checklists Designed Along the Lines of Aviation Checklists Will FAIL

Semi-closed environment	Open environment
Two people (most often)	Many people, number can vary over time
Same specialty – same tasks	Different specialties – different tasks
Highly ordered and proceduralized	Ordered but not so proceduralized
Medium-to-high level of predictability	Medium-to-low level of predictability
Medium variability across aircraft	High variability across patients
Minimal differences in equipment used daily	Can be moderate differences in equipment used daily

Burian, et al, submitted for publication

THE UNIVERSITY OF CHICAGO MEDICINE

These Quick Reference Guides are not a Seatbelt, they are a GPS

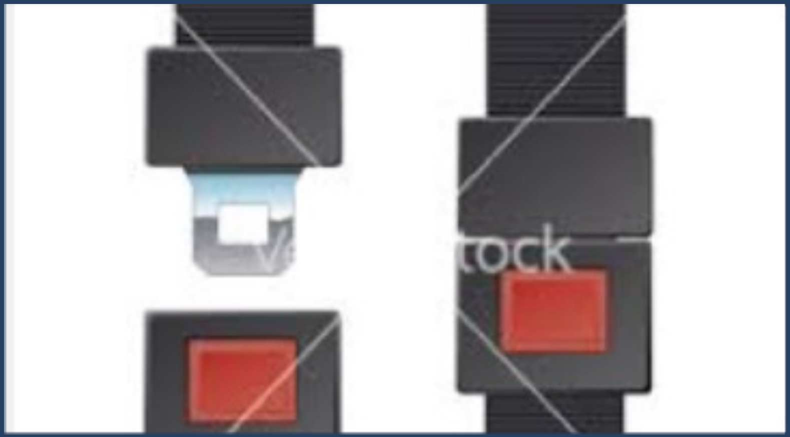

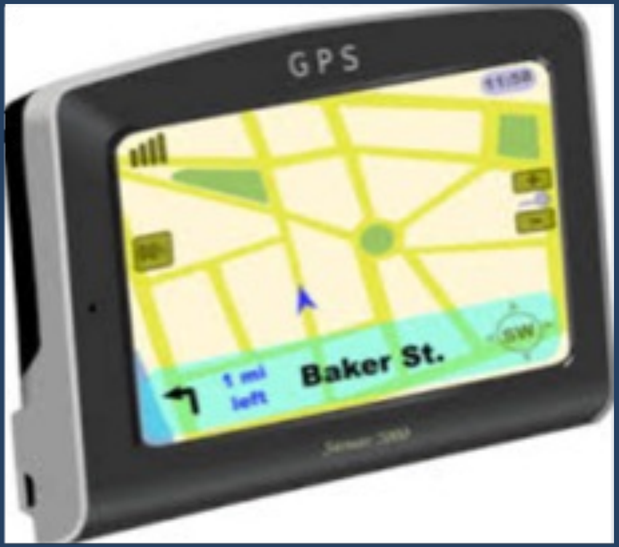

THE UNIVERSITY OF CHICAGO MEDICINE

What Doesn't Work?

1) Aid doesn't match purpose and environment

2) Bad content and poor design

3) cultural barriers: professional, institutional

4) insufficient or non-existent induction and/or training

How to fix: ask yourself

1) Why needed and what change do we want?

2) Content serves purpose? Does design facilitate, not hinder?

3) Clear purpose? Did you invite members of all potential user groups to create and implement?

4) What training is needed?

Burian, et al, submitted for publication

THE UNIVERSITY OF CHICAGO MEDICINE

Where?

Why?

How?

Who?

THE UNIVERSITY OF CHICAGO MEDICINE

STEP BY STEP VERSUS SAMPLING

8 Hemorrhage

Acute massive bleeding

START

1. **Call for help and a code cart**
   - **Ask**: *"Who will be the crisis manager?"*
2. **Open IV fluids** and **assess for adequate IV access**
3. **Turn FiO_2 to 100%** and **turn down volatile anesthetics**
4. **Call blood bank**
   - Activate massive transfusion protocol
   - Assign 1 person as primary contact for blood bank
   - Order blood products (in addition to PRBCs)
     - 1 FFP : 1 PRBC
     - If indicated, 6 units of platelets
5. **Request rapid infuser** (or pressure bags)
6. **Discuss management plan** between surgical, anesthesiology, and nursing teams
7. **Call for surgery consultation**
8. **Keep patient warm**
9. **Send labs**
   CBC, PT/PTT/INR, fibrinogen, lactate, arterial blood gas, potassium, and ionized calcium
10. **Consider**...
    - Electrolyte disturbances (hypocalcemia and hyperkalemia)
    - Uncrossmatched type O blood if crossmatched blood not available
    - Damage control surgery (pack, close, resuscitate)
    - Special patient populations (see considerations below)

DRUG DOSES and treatments

HYPOCALCEMIA treatment

Give calcium to replace deficit (calcium chloride or calcium gluconate)

HYPERKALEMIA treatment

1. Calcium gluconate<br>- or -<br>Calcium chloride	• 30 mg/kg IV<br><br>• 10 mg/kg IV
2. Insulin	• 10 units regular IV with 1–2 amps D50W as needed
3. Sodium bicarbonate if pH <7.2	• 1–2 mEq/kg slow IV push

SPECIAL PATIENT POPULATIONS

OBSTETRIC:
- Empirical administration of 1 pool of cryoprecipitate (10 cryo units)
- Check fibrinogen (goal is > 100 mg/dL)
  If first fibrinogen level < 100 mg/dL, order 2 more pools of cryoprecipitate

TRAUMA:

Give <u>either</u>...
- Antifibrinolytic tranexamic acid: 1000 mg IV over 10 minutes followed by 1000 mg over the next 8 hours
  – *or* –
- Aminocaproic acid: 4–5 g in 250 mL NS/RL IV over first hour followed by a continuing infusion of 1 g in 50 mL NS/RL IV per hour over 8 hours

NON-SURGICAL UNCONTROLLED BLEEDING despite massive transfusion of PRBC, FFP, platelets and cryo:
- Consider giving Recombinant Factor VIIa: 40 mcg/kg IV
  – Surgical bleeding must first be controlled
  – **use with CAUTION** in patients at risk for thrombosis
  – **DO NOT use** when PH is < 7.2

8

All reasonable precautions have been taken to verify the information contained in this publication. The responsibility for the interpretation and use of the materials lies with the reader. Revised July 2013 (072413.1)

THE UNIVERSITY OF CHICAGO MEDICINE

Burian, et al, submitted for publication

What Doesn't Work?

1) Aid doesn't match purpose and environment

2) Bad content and poor design

3) cultural barriers: professional, institutional

4) insufficient or non-existent induction and/or training

How to fix: ask yourself

1) Why needed and what change do we want?

2) Content serves purpose? Does design facilitate, not hinder?

3) Clear purpose? Did you invite members of all potential user groups to create and implement?

4) What training is needed?

Burian, et al, submitted for publication

THE UNIVERSITY OF CHICAGO MEDICINE

What Doesn't Work?

How to fix: ask yourself

1) Aid doesn't match purpose and environment

1) Why needed and what change do we want?

2) Bad content and poor design

2) Content serves purpose? Does design facilitate, not hinder?

3) cultural barriers: professional, institutional

3) Clear purpose? Did you invite members of all potential user groups to create and implement?

4) insufficient or non-existent induction and/or training

4) What training is needed?

Burian, et al, submitted for publication

QUICK REFERENCE GUIDE – STEP BY STEP

1) Organization
2) Structure
3) Team
4) Items
5) Simulate
6) Complexity

THE UNIVERSITY OF CHICAGO MEDICINE

University of Chicago Department of Anesthesia and Critical Care

THE UNIVERSITY OF CHICAGO

ADULT Crisis Quick Reference Guide/Cognitive Aid

Air Embolism	1
Anaphylaxis	2
Bradycardia/Pacing	3
Cardiac Arrest	4
Hyperkalemia	5
Increased Intracranial Pressure	6
Local Anesthetic Toxicity	7
Malignant Hyperthermia	8
Tension pneumothorax	9
Transfusion: Massive Hemorrhage	10
Transfusion Reactions	11
MAPS of DACC Locations	12-15

Adapted for local use from resources authored by The Society for Peds Anesthesia, Ariadne Labs, ASRA, and MHAUS

Call attending, call for help!

Anest. Coordinator dial 64470 page #4470

Notify surgeon.

***This reference is meant to serve as a memory aid and is not intended to proscribe care. It does not replace your clinical judgment.

Revision July 19, 2016

3 Bradycardia/Pacing ADULT

VERIFY DX – STABILIZE PATIENT

- Verify Bradycardia: ↓ HR ↓ BP with clinical evidence of poor perfusion
- Switch to 100% O_2, evaluate ventilation
- If ΔHR & ΔBP mild, consider
  - glycopyrrolate 0.4 mg or
  - atropine 0.4 mg

TREATMENT

Cause	Action
Surgical stimulation	▪ If laparoscopic, de-sufflate<br>▪ If not laparoscopic, stop surgery
Myocardial ischemia	▪ O_2, NTG, morphine, beta-blockers (when tolerated), if OK ASA, clopidogrel
Beta-blocker overdose	▪ Glucagon 3-10 mg IV, then 0.07 mg/kg/hour IV infusion
Ca-channel blocker overdose	▪ Calcium chloride 1 mg IV *or*<br>▪ Calcium gluconate 1-2 mg IV<br>▪ If ineffective, Glucagon at above doses
High spinal	▪ See CHKLST 7 (LAST)
External/internal pacer failure	▪ Contact electrophysiology (pager #4118)
Cardiac arrest	▪ See CHKLST 4 (Arrest)

CRISIS MANAGEMENT (IF SEVERE)

- Notify surgeon, call for help and code cart. Check pulse
- If no pulse:
  - Start chest compressions
  - Give epinephrine 1 mg IV
  - Consider transcutaneous pacing (see inset)
  - If cardiac arrest, See CHKLIST 4 (Arrest)
  - Consider ECMO (pager #2871)

DRUG / DOSAGE SUMMARY

- **Atropine** 0.4 mg
- **Calcium chloride** 1 mg IV
- **Calcium gluconate** 1-2 mg IV
- **Epinephrine** 1 mg IV
- **Glucagon** 3-10 mg IV, then
  0.07 mg/kg/hour IV infusion
- **Glycopyrrolate** 0.4 mg

Instructions for PACING

1. Call for Pacer/Defibrillator
2. Place pacing ECG electrodes AND pacer pads on chest.
3. Turn monitor/defibrillator ON, set to PACER mode
4. Set PACER RATE (ppm) to desired rate/min (80).

   Can be adjusted up or down based on clinical response once pacing is established
5. Increase the PACER OUTPUT (mA) until electrical capture

   Pacer spikes aligned with QRS complex. Threshold normally 65-100mA
6. Set mA to 10mA above this level
7. Confirm pulse is present. If not, repeat steps 4-5

QUICK REFERENCE GUIDE - STEP BY STEP

1) Organization
2) Structure
3) Team
4) Items
5) Simulate
6) Complexity

THE UNIVERSITY OF CHICAGO MEDICINE

QUICK REFERENCE GUIDE - STEP BY STEP

1) Organization
2) Structure
3) Team
4) Items
5) Simulate
6) Complexity

THE UNIVERSITY OF CHICAGO MEDICINE

2 Anaphylaxis ADULT

VERIFY DX – STABILIZE PATIENT

- Verify Anaphylaxis, may have: Rash, bronchospasm, hypotension
- Switch to 100% O_2, evaluate ventilation
- Remove suspected trigger(s)
  - If latex is suspected, thoroughly wash area, have surgeons change to non-latex gloves
  - If HYPOtensive, turn off anesthetic agents

TREATMENT

Cause	Action
Decreased intravascular volume	▪ NS or LR 1 L IV, **rapidly**, may need 5-10 L
Hypotension<br>Bronchospasm	▪ Epinephrine 10-100 mcg IV/IO, as needed, may need infusion 0.02-0.2 MICROgrams/kg/min
Hypotension refractory to epinephrine	▪ Vasopressin 1-2 units IV
Bronchospasm	▪ Albuterol 4-10 puffs or more as needed
Mediator release	▪ Hydrocortisone 100 mg IV
Histamine release	▪ Diphenhydramine 25-50 mg IV, famotidine 20 mg IV

Differential

Fat, thrombotic, cement, amniotic fluid embolus	▪ See CHKLST 1 (embolus)
Sepsis	▪ Support BP, antibiotics
Myocardial Ischemia	▪ Morphine, oxygen, aspirin, NTG, beta-blocker, clopidrogel, heparin

DRUG / DOSAGE SUMMARY

- **Epinephrine** 10-100 mcg IV/IO, as needed, may need infusion 0.02-0.2 MICROgrams/kg/min
- **Vasopressin** 1-2 units IV
- **Albuterol** 4-10 puffs or more if needed
- **Hydrocortisone 100** mg IV
- **Diphenhydramine** 25-50 mg IV
- **Famotidine** 20 mg IV

Common CAUSATIVE Agents

- Neuromuscular blockers
- Latex
- Chlorhexidine gluconate prep solution
- IV colloids
- Antibiotics

CRISIS MANAGEMENT (IF SEVERE)

- Notify surgeon, call for help and code cart. Check pulse
- If no pulse:
  - Start chest compressions
  - Give epinephrine 1 mg IV/IO
  - If cardiac arrest, See CHKLIST 4 (Arrest)
  - Consider ECMO (pager #2871)

Anaphylaxis ADULT

Where is the MH cart located in the CCD?

1) In the anesthesia supplies room
2) In the hall, by the coordinator's office
3) In the central core outside of OR 18
4) In the cardiac core outside of OR 1

THE UNIVERSITY OF CHICAGO MEDICINE

MH hotline 1-800-644-9737

8 Malignant Hyperthermia ADULT

VERIFY DX – STABILIZE PATIENT

- Verify MH, may have: ↑ Temp ↑ HR ↑ CO_2 acidosis
- Notify surgeon, call for help, MH cart (CCD: in core next to OR 18, Comer: in anesthesia workroom)
- Stop procedure, if possible
- Stop volatile and succinylcholine and transition to non-triggering anesthetic
- Give **dantrolene** 2.5 mg/kg IV every 5 min until symptoms resolve
  - Dantrium/Revonto: Assign dedicated person to mix these formulations of dantrolene (20 mg/vial) with 60 mL non-bacteriostatic sterile water
  - Ryanodex: 250 mg is mixed with 5 mL non-bacteriostatic sterile water

TREATMENT

Cause	Action
Volatile in circuit	▪ Attach charcoal filters to inspiratory and expiratory limbs
Acidosis	▪ Hyperventilate with FiO2 100% flow >10 L/min<br>▪ Give sodium bicarbonate 1-2 mEq/kg IV for suspected metabolic acidosis; maintain pH > 7.2
Temperature > 39º C	▪ Apply ice externally to axilla, groin and around head<br>▪ Infuse cold saline intravenously<br>▪ Lavage body cavities (NG, foley) with cold water<br>▪ Stop cooling when temperature < 38º C
Hyperkalemia	▪ Calcium gluconate 1-2 g IV or calcium chloride 1 g IV<br>▪ Regular insulin 10 units and dextrose 25-50g

DRUG / DOSAGE SUMMARY

- **Dantrolene** 2.5 mg/kg IV
- **Sodium bicarbonate** 1-2 mEq/kg IV
- **Calcium gluconate/chloride 1-2** g IV
- **Insulin** 10 units and **dextrose** 25-50 g

TREATMENT (cont.)

Cause	Action
Dysrhythmias	▪ Do NOT use calcium channel blocker; standard antiarrhythmics are acceptable
Need for monitoring labs	▪ Serial ABGs or VBGs, electrolytes, serum CK, serum/urine myoglobin, coagulation
Need to maintain urine output	▪ Place foley catheter to monitor urine output for goal >1mL/kg/hour<br>▪ Consider 20mg furosemide or 0.25g/kg mannitol

CRISIS MANAGEMENT (IF SEVERE)

- Notify surgeon, call for help and code cart. Check pulse
- If no pulse:
  - Start chest compressions
  - Give epinephrine 1 mg IV/IO
  - If cardiac arrest, See CHKLIST 4 (Arrest)
  - Consider ECMO (pager #2871)

QUICK REFERENCE GUIDE - STEP BY STEP

1) Organization
2) Structure
3) Team
4) Items
5) Simulate
6) Complexity

THE UNIVERSITY OF CHICAGO MEDICINE

Case: 64 yo for parathyroidectomy
PMH: Parathyroid adenoma. Hypotension during prev surg.
Preop vitals: BP 130/87, HR 68, SaO2 98%, RR 11.

Anesthesia: Standard induction with midazolam, fentanyl, propofol, and rocuronium

Intraoperatively: Pt develops hypotension and bronchospasm and peak airway pressures of 48 cmH2O

What would you do next?

The BP then drops to 60/40, HR 80

Now what would you do?

THE UNIVERSITY OF CHICAGO MEDICINE

2 Anaphylaxis ADULT

VERIFY DX – STABILIZE PATIENT

- Verify Anaphylaxis, may have: Rash, bronchospasm, hypotension
- Switch to 100% O_2, evaluate ventilation
- Remove suspected trigger(s)
  - If latex is suspected, thoroughly wash area, have surgeons change to non-latex gloves
  - If HYPOtensive, turn off anesthetic agents

DRUG / DOSAGE SUMMARY

- **Epinephrine** 10-100 mcg IV/IO, as needed, may need infusion 0.02-0.2 MICROgrams/kg/min
- **Vasopressin** 1-2 units IV
- **Albuterol** 4-10 puffs or more if needed
- **Hydrocortisone 100** mg IV
- **Diphenhydramine** 25-50 mg IV
- **Famotidine** 20 mg IV

TREATMENT

Cause	Action
Decreased intravascular volume	NS or LR 1 L IV, **rapidly**, may need 5-10 L
Hypotension Bronchospasm	Epinephrine 10-100 mcg IV/IO, as needed, may need infusion 0.02-0.2 MICROgrams/kg/min
Hypotension refractory to epinephrine	Vasopressin 1-2 units IV
Bronchospasm	Albuterol 4-10 puffs or more as needed
Mediator release	Hydrocortisone 100 mg IV
Histamine release	Diphenhydramine 25-50 mg IV, famotidine 20 mg IV

Differential

Fat, thrombotic, cement, amniotic fluid embolus	See CHKLST 1 (embolus)
Sepsis	Support BP, antibiotics
Myocardial Ischemia	Morphine, oxygen, aspirin, NTG, beta-blocker, clopidrogel, heparin

Common CAUSATIVE Agents

- Neuromuscular blockers
- Latex
- Chlorhexidine gluconate prep solution
- IV colloids
- Antibiotics

CRISIS MANAGEMENT (IF SEVERE)

- Notify surgeon, call for help and code cart. Check pulse
- If no pulse:
  - Start chest compressions
  - Give epinephrine 1 mg IV/IO
  - If cardiac arrest, See CHKLIST 4 (Arrest)
  - Consider ECMO (pager #2871)
