# Supplementary material for: Why are critical event checklists not always used in the perioperative setting?: A retrospective survey
Source: PLoS One. 2025 Feb 28;20(2):e0314774. doi: 10.1371/journal.pone.0314774 (PMC11870359; doi:10.1371/journal.pone.0314774)
Supplement: S4 File — (DOCX) [file pone.0314774.s004.docx]

**Narrative Reasons for Aid Non-Use**

***if part of the narrative response, individual’s names (e.g. Dr. ___ did ___ ) and case details taken out for privacy**

**INITIAL (if multiple dx were cited, the most specific dx was chosen)**

**Cognitive Aid Non-Use**

**Air embolus:**

- Not available at the time.

**Anaphylaxis:**

- Knew what to do
- No cognitive aid for anaphylaxis
- Not available, time limitation
- Did not know where to find it
- Not available
- Did not feel the need - knew the tx for anaphylaxis already and help came in <30 seconds
- None available
- (did not specify)

**Bradycardia:**

**-**None available

**-** I knew which treatment was required without cognitive help

-Not available

- HR - 70s, decreased heart block for <3 sec. Gave glycophyrrolate 0.4 mg IV, HR increased to 110s. It was no fast, that the situation was remedied

**-**easy fix

- I did not use before mentioned aids, but I recently had sim w/[redacted] in June and the checklist her and [redacted] have worked on helped a great deal with events above

-regular old cardiac arrest. I helped to attempt transvenous pacing, which did not work and the patient expired. A cognitive aid such as a pre-packaged kit for transvenous pacing would have helped as we struggled to find the wire, the corresponding pacer box, etc.

-not immediately available

-Atropine is step 1

-Unavailable. Straightforward therapy + inciting cause.

-treatment was obvious - atropine

**Cardiac arrest:**

- Dr. [redacted] was my aid
- Not available
- Not readily available
- The code team leader was running the event sequence
- Cardiac arrest happened in the PACU during TEE. Because TEE images were on the screen it becameimmediately obvious
- I was not the team leader/. came in to assist with blood infusion.
- Came in to help with CPR.
- Was not there at the beginning of critical event.
- Everyone in the room new {sic} what to do
- We technically used the ACLS protocol, but we did not pull out the aid and follow it strictly.
- We were summoned to a CART for cardiac arrest and were simply requested to intubate the patient.
- Not immediately available
- None immediately available and the event was very short. I came in towards the end of this event. The patient had been in PEA arrest; however, had a pulse by the time entered the room. Patient was quickly transported to ICU after the event.
- ACLS protocols not immediately at hand, but verbally prompted
- No need
- No need
- Offsite location, fast paced rapid response, unaware of how to access one offsite.
- not available (or I was not aware of it)
- N/A

**Hyperkalemia:**

-Did not need to

- Cognitive aid was not consulted because the cause of arrhythmia was reasonably well known, and management was adequate to resolve the problem.

-New {sic} basic steps to take

-standard management initiated

-Calcium and Glucose/Insulin did the trick

-Did not have one

**Hypertension:**

**-**Attending was notified and present. Proper treatment was discussed and administered

**Hypotension:**

**-**None available

- Situation was hypotension clearly due to bleeding in a post-cardiac surgery patient.

-Not immediately available

-No need

-Not readily available

- Checklist may be helpful in rare events; we treat hypotension daily.

-Busy intervening (blousing fluid, hanging blood, administering pressors). Intervening quickly w/ little time to access a cognitive aid.

-None available

-Not necessary as TEE immediately established dx. (LV and RV dysfxn)

-TEE insertion immediately established cardiac tamponade

-Unavailable. Common problem and quick albeit non-specific fixes.

**Hypoxia:**

**-** Multiple people helping manage patient

**-** Not available

**-** Unaware of cognitive aid for acute hypoxic respiratory failure

- Patient was ICU already intubated and became hypoxic from a large mucous plug. There was no relevant cognitive a that we could use other than ACLS, which was already being employed by the cart team

- Didn't have a chance to - also patient became hypoxic -> brady -> arrest

- None available

- acls, known algorithm, if not known would have referred

- I already knew the next appropriate steps

-Re intubated patient

- We did not have one available to my knowledge for handling intraop hypoxia. We ran through a mental checklist of handling hypoxia, particularly since this was a thoracic case and we were having difficulty ventilating.

- many cognitive aids in this situation such as PALS or ACLS do not apply the same in the OR in many cases of bradycardia and would not have been useful

- emergency management

-No need

-Not readily available

- Called overhead to [redacted location]. Patient with low sats and slowing HR. Prone sedation, we turned supine, masked and gave atropine for brady. No aids needed.

-not in charge, only helping, straightforward issue that needed addressing

-easy fix

- new CA-1, needed more experienced person at airway

- stressful, limited time to read/address

- I did not use before mentioned aids, but I recently had sim w/Dr. [redacted] in June and the checklist her and Dr. [redacted] have worked on helped a great deal with events above

-The issue at hand was re-orienting the treatment team and re-establishing a line of communication.

-Not needed, knew cause, pt improving

-Already had 3 providers (me, fellow, and attending) who worked well as a team to manage the patient

-(no reason given)

-Trying to identify the main problem with many different vitals becoming unstable at once.

-(did not specify)

-3 reasons for hypoxia

-I wasn’t aware of any aids that I could use

-Unavailable. Already familiar

**Increased ICP:**

- neuro icu attending was in charge and had things under control

**Malignant Hyperthermia:**

- Not available
- I was just getting supplies that were needed when asked.
- Did not feel I needed it

**Massive Hemorrhage:**

- None available
- I had my attending with me and plenty of help
- Not available
- Did not need to
- Did not need to
- Did not need to
- Did not need to
- Patient developed massive hemorrhage after [redacted] procedure. MTP was activated. All necessary personnel was immediately available, and their performance was excellent and timely.
- Multiple teams present to help with gathering resources and coming up with plan. Did not think to use manual
- I felt I knew what to do and did not have time to go looking for an appropriate cognitive aid
- Didn't think was needed
- i was not the primary
- i don’t recall because I was not the primary
- None available
- maybe could have, did not stop to think about it between transfusing and calling surgery
- Not immediately available
- No need
- Not readily available
- Did not feel I needed it
- We got control of the situation quickly
- familiar with management of hemorrhage
- Anesthesiologist ran case
- (did not specify)
- Unavailable to my knowledge

**Myocardial Ischemia:**

- none available
- previously memorized treatment algorithm
- did not need it. the patient had died. we were going for ECMO - unsuccessfully

**Pulmonary embolus:**

- None readily available at bedside
- Multiple attendings and personnel involved with collaboration of how to handle situation
- No time
- ACLS – if I did not know it I would refer to it
- It was straightforward cardiac arrest with identifiable intracardiac thrombi. (and liver transplant)
- (did not specify)

**Pulmonary hypertension:**

- Attending present
- again, probably could have, between transfusing, starting vasoactives and coordinating services did not stop to think about using a cognitive aid

**Tachycardia:**

**-** Event resolved rapidly with initial treatment

**Tension Pneumothorax:**

-knew what to do

**Unexpected difficult airway:**

- none available
- Unanticipated difficult airway in an elderly patient with septic knee after knee replacement. DL and videolaryngoscope were not helpful, airway was not seen. Two attending anesthesiologists came to help, and patient was intubated with the use of the bronchoscope. Since time was of essence, cognitive aid was not used.
- Not immediately available
- I felt I knew what to do and did not have time to go looking for an appropriate cognitive aid
- Not available
- Not immediately available
- Already familiar with treatment algorithms
- Things happening too fast to look up, knew what to do
- NA
- NA
- There was not enough time
- Not readily available
- This could also be a yes answer technically, since I had to remind the team leader that non-synchronized cardioversion is recommended for unstable SVT. The team also received advise about the difficult airway algorithm, but that came from other helpers. But I didn't "pick up" a cognitive aid.
- ENT pt, ENT attending and I used FOB to secure airway
- Didn’t need one
- Wasn’t readily available aka I’d have to dig through the drawer
- Wasn’t readily available aka I’d have to dig through the drawer
- didn't need it .I contributed the mixed strategy of aintree catheter and LMA and IV access (U/S guided) which fixed the situation. Arguably we were utilizing the difficult airway algorithm which is intuitive AND well known to us
- familiar with ASA algorithm
- didn’t have any
- “we knew what to do”
- (did not specify)
- Managing airway
- I was aware what needs to be done
- I knew the algorithm by heart

**-**We loosely followed the difficult airway algorithm. DL then glidescope then bougie then successful

**-**Familiarity with difficult airway algorithm. Attending immediately available.

-not specified

**Other:**

**Post-op respiratory failure:**

- None available

**emergent c-section of a baby with a neck mass/difficult airway**

**-** plan was already in place to call peds ENT attending from home

**neck hematoma with respiratory compromise in PACU**

- I didn't need one. The patient's incision had already been opened by the time I got there and we knew the patient needed to be intubated.

**NA**

**-**na

**Bronchospasm:**

**-**Is there a cognitive aid for bronchospasm/laryngospasm?

- not helpful to read something when I should be taking care of the patient

**Morbidly obese patient requiring EUA. It wasn't necessarily an unanticipated difficult airway, but she ended up being a harder than expected DL, requiring LMA:**

- This was a straightforward difficult DL, then worsening mask. It was not unanticipated per se (hence me already being in the room). When DL had failed x2, we placed an LMA and proceeded with the case.

**PACU apnea:**

- Not helpful to read something when I should be mask ventilating pt

**Laryngospasm:**

**-** Not helpful to read something when I should be mask ventilating pt

**Uncooperative pt with ongoing NSTEMI who was uncooperative then coded:**

- Pt needed to be intubated. Did not need a checklist.

**Extubation during case:**

**-**Leaning on attending for how he would like to reestablish airway.

**Spinal cord injury:**

**-**I was aware of what needed to be done

**Seizure:**

-I did not see one

**bilateral pleural effusion:**

-We weren’t sure what the exact problem was

**Fat embolus:**

-unavailable to my knowledge

**FOLLOW-UP**

**Cognitive Aid Non-Use**

**Anaphylaxis:**

-Forgot it was in drawer.

-Didn’t Need It

-Acute situation I knew how to manage and needed immediate treatment. I later looked at the cognitive aid to make sure I didn't miss anything.

-Attending quickly provided guidance

-Differential diagnosis between anaphylaxis and isolated bronchospasm.

-For the anaphylaxis, honestly did not think of it

**Air Embolus:**

-Event lasted <1 minute. Rapid resolution with pressors, chest thump

**Bradycardia:**

-Pt responded to treatment

-i did not need it

-reason for bradycardia was vagal and apparent and occurred quickly. so easily treated with atropine.

-{no explanation given}

-known etiology and treatment plan

-{no explanation given}

- Familiar with management

- Acute situation that almost required ACLS. I treated quickly with atropine and resolved. The situation was extra calcium blocker. The cognitive aid covered this.

- too easy to deal with - zI know the causes by heart

-I knew what I needed to do

- Peds, gave atropine, quickly resolved

-(bradycardia x 2) Pt responded to our initial interventions

**Cardiac arrest:**

-Would've likely used a cognitive aid had the patient not responded so quickly.

-I did not need it

-comfortable with needs of the moment

-situation was totally unexpected. rapid onset and progression hypotension/brady to arrest. didn't think of it in the moment.

-Not available

-The attending was present and knew what to do

-reflex response

-Was not part of team

-Not aware of it; more focused on helping

- Forgot/Too Busy w/ Patient Care

- Already comfortable with how to deal with situation.

- Attending was directing case and knew the next steps in management

**Difficult Airway:**

-We utilized steps of the difficult airway algorithm to troubleshoot the airway and were able to successfully intubate prior to use of a cognitive aid. The situation never became an emergency because we were able to ventilate adequately. Both providers were well-versed in the difficult airway algorithm and were able to appropriately follow the airway without a cognitive aid.

-did not think at the time of it

-{no explanation given}

-Did not require.

-I was concentrating on establishing a secure airway

-i did not need it

-experience easily sufficed

-No time, I was already escalating the care and had called for help.

-Not necessary as AW was being secured with FOB.

-{no explanation given}

-did not need it

-People in the room seemed to always have an idea for the next logical step. No one ever just stopped and didnt know what to do next

-So time sensitive

-Difficult nasal intubation with Glide visualization. Patient never desaturated (was spontaneously ventilating throughout), required multiple attempts to pass ETT and developed epistaxis but did not follow DAA.

-Next step already known

-I followed different airway algorithm in my head

-no time as I had to immediately take over from CA3 who was unable to intubate

with a MAC 3 in setting of acute vomiting

-Did not need one

-We used difficult airway algorithm

-no time

-ASA difficult airway algorithm memorized

-Attending and I folllowed difficult airway algorithm from memory (x2 events)

-Did not feel one was necessary for event. Knew next steps/interventions.

-Diffi airway algorithm in my mind's eye

-did not need at the time went down difficult airway algorithm

-Not aware of it

-I don't think there was one available, didn't think about using one

-ASA Algorithm and Vortex approach applied, visual aid would have been helpful, but was able to ventilate and move down the algorithm with success

-Thought of difficult airway algorithm

-I did not have free hand available

-Several attendings were able to use difficult airway algorithm

**Massive Hemorrhage:**

-Forgot it was in drawer. Wish I had remembered.

-{no explanation given}

-Did not require

-{no explanation given}

-We knew the source of the bleeding. We didn't feel it was necessary to use a cognitive aid since the anesthesia team knew how to diagnose and treat.

-{no explanation given}

-{no explanation given}

- People jumped in and everyone played a role immediately

-Already knew my next step. Also momentary decision had to be made and there was not time to consult a cognitive aid.

-This was a post op bleed, MTP was used. Tx was 9 U PRBC, 7 FFP, with multiple POC tests

-too much experience with it

-The management observed was appropriate

-Forgot/Too Busy Performing Patient Care

-attending in charge was directing action

-Massive transfusion protocol in place in L & D

-Busy checking and giving blood

-x >4 massive hemorrhage. Expected high blood loss surgery, plan in place

-Not required. Transfusion initiated, course of hemorrhage known

-my attending and senior residents were giving me instructions on what to do

**Hyperkalemia:**

-attending and CRNA caring for the patient knew the correct actions to take in management of a hyperkalemic patient

-no need

-I knew what I needed to do

-was familiar with protocol

**Hypertension:**

**-**Again forgot we had it.

**Hypotension:**

-We knew the cause of hypotension- iatrogenic secondary to medications.

-Forgot.

-I followed by own planvto good effect

-It became clear that patient experienced intra-abdominal bleeding, and we went to the OR. Massive transfusion protocol was initiated.

-{no explanation given}

-experience easily sufficed

-There was more blood loss than expected and a contaminated wound, so was I was fairly certain of the cause and how to treat (once we finally received the blood we ordered).

-TEE diagnosed etiology and guided treatment.

-busy giving fluid, albumin, pressors, ca, calling attending

-not available

-Did not think I needed one, quick resolution of problem

-didn't think to do it cuz we were too busy taking care of the pt

-the monitors indicated the patient was fluid responsive in the first episode, we obtained an echo in the second.

-peri-cardiac arrest, attending already in room, TEE probe already in place, cognitive aid not needed

-only used after hypotensive episode to double check my plan. was useful that it was a quick checklist

-I knew what I needed to do

-I did not think there was a specific cognitive aide for the situation.

-I was completing other tasks

-Not required. proceeded with administering fluids/pressors/resuscitation.

**Hypoxia:**

**-** I knew the ddx and responded accordingly. PE wound up being what occurred.

**-** Quickly diagnosed etiology

**-** experience easily sufficed

**-** I went through the differential in my head and checked the ETT, patient, machine and fixed the issue.

| - known etiology, treatment plan |
| --- |
| - It was a laryngospasm event and I know the way to treat this without having to consult any cognitive aids - I am unaware of a cognitive aid that is helpful for the treatment or work-up hypoxia. - Cause of hypoxia was known - large A-a gradient due to pulmonary aspiration of massive epistaxis. Poorly tolerated apnea for his trach and bronch (desats to mid 50s with <1 minute of apnea) - desaturation quickly upon extubation, tending to patient - no time |
| - x3 - 1,2) did not feel one was necessary. knew cause/next steps (pediatric laryngospasm) 3) worked through ddx with resident, knew cause/etiology although I did show the DACC cognitive aid at the end to familiarize resident with it |
| - Resolved promptly. And it was mechanical issue shoulder strap strangling pt. I doubt aid would help. |
| - laryngospasm; hands full taking care of pt; by the time help arrived laryngospasm broken, pt then intubated, problem solved - I was getting ready to intubate – patient had an LMA   **Increased ICP:**   - familiar with all the steps since this is my subspecialty   - did not think to use it  **Myocardial infarction:** |
|  |
| -Forgot  -Occurred post-op in the ICU  -Was not mypatient, others were in charge  -no need.  -Attending in room during event  **Other:**  **Serotonin syndrome:**  -Not available and honestly I did not know what it was until after the patient was in the ICU and the residents and I did some digging.  **expected difficult airway; delayed diagnosis infiltrated iv with hypotension:**  -we diagnosed/fixed situation fairly rapidly; also experience sufficed |
| **gas pipeline concern:**  -there isn't one  **laryngospasm:**  -attending was present, knew what to do. situation was fixed by the time I entered the room  **Shock:** |
|  |
| **-**Not required. Proceeded with resuscitation. |
