# Supplementary material for: Why are critical event checklists not always used in the perioperative setting?: A retrospective survey
Source: PLoS One. 2025 Feb 28;20(2):e0314774. doi: 10.1371/journal.pone.0314774 (PMC11870359; doi:10.1371/journal.pone.0314774)
Supplement: S5 File — (DOCX) [file pone.0314774.s005.docx]

Supplemental Content: Events for which the cognitive aid was accessed and reasons for aid use

| Provider | Event | What Prompted Aid Use? | At What Point Was Aid Accessed? |
| --- | --- | --- | --- |
| Baseline | | | |
| Faculty | Massive Hemorrhage | Just making sure things got done | After the initial response -- *to make sure we had not forgotten anything. |
| CRNA | MH and cardiac arrest | Already in use when came in room to help | N/A |
| CA-3 | Air Embolus Pulmonary Embolus Unexpected Difficult Airway Massive Hemorrhage Hypotension Hypoxia | Knowing the importance of the protocol | Very beginning |
| Fellow | Fellow, Unexpected Difficult Airway | Didn't pick it up, just had studied it in general | Didn't pick it up, just had studied it in general |
| CA-2 | cardiac arrest | It wasn't a physical card or anything, just the ACLS protocol with which we're all familiar | It wasn't a physical card or anything, just the ACLS protocol with which we're all familiar |
| CRNA | MH | I believe one of the attendings who came into the room began using a list supplied on the MH cart | *After the initial treatment of the event. We had already turned off the agent, increased flows, etc.., starting giving dantrolene, and cooling. |
| CA-2 | OB hemorrhage | It was nearby and had pertinent info | *Disagreement with attending about next steps |
| Fellow | Cardiac arrest | Memorized | memorized |
| Faculty | Hyperkalemia | (not answered) | (not answered) |
| CRNA | Hypotension | It was immediately available | Right away |
| CA-1 | Massive Hemorrhage | Knowing that it was available | At sign of excessive hemorrhage. |
| CA-3 | Unexpected difficult airway | I had it memorized in my head | after first airway attempt failed |
| Provider | Event | What Prompted Aid Use? | At What Point Was Aid Accessed? |
| Follow-Up (After Cognitive Aid Training) | | | |
| Faculty | Anaphylaxis | The need for quick action | immediately |
| CRNA | Anaphylaxis | clinically relevant | beginning |
| CA-3 | Cardiac Arrest | Not to miss any possible cause. | *After first ROSC. |
| CA-1 | Cardiac Arrest | ____attending had it | *Towards the end/[next response] just to make sure we hadn’t missed anything |
| CRNA | Cardiac Arrest | (no answer) | (no answer) |
| CA-1 | Cardiac Arrest | known to be available | 1st 2 minutes of code |
| CRNA | Massive Hemorrhage | I usually check it when I have a free moment to make sure we have done everything. I do not use it concurrently. | See above: *Once the first several steps had been done and the patient was stable I picked it up to make sure we had not missed anything. |
| Faculty | Massive Hemorrhage | Easy to find | Beginning |
| CA-2 | Massive Hemorrhage | ______had been talking about that cognitive aid at lecture the morning of the event. I specifically knew it had the number for the blood bank available! | *After notifying my attending and adjusting O2/fluids. |
| Faculty | Hyperkalemia | dosing | immediately |
| CRNA | Hypertension | (no answer) | (no answer) |
| CRNA | Hypotension | (no answer) | (no answer) |
| CRNA | Hypoxia | (no answer) | (no answer) |
| Faculty | Tension Pneumothorax | the need for rapid treatment of hypoxia | *after we treated the patient with PEEP and this seemed to not help, making pneumothorax a more likely diagnosis |
| Faculty | Massive Transfusion | Primary anesthesiologist utilized to mobilize resources including IV access and product administration | Beginning |
| Legend: *aid accessed after initial critical steps performed | | | |
